# Antibacterial activity of inverse vulcanized polymers

**DOI:** 10.1021/acs.biomac.1c01138

**Published:** 2021-11-16

**Authors:** Romy A. Dop, Daniel R. Neill, Tom Hasell

**Affiliations:** 1 Department of Chemistry, University of Liverpool, Liverpool L69 7ZD, United Kingdom; 2 Department of Clinical Infection, Microbiology and Immunology, Institute of Infection, Veterinary and Ecological Sciences, University of Liverpool, Liverpool, United Kingdom

**Keywords:** Sulfur polymer, inverse vulcanization, antibacterial, *Staphylococcus aureus*, *Pseudomonas aeruginosa*, biofilm

## Abstract

Inverse vulcanization is a bulk polymerization method for synthesizing high sulfur content polymers from elemental sulfur, a by-product of the petrochemical industry, with vinylic comonomers. There is growing interest in polysulfides as novel antimicrobial agents due to the antimicrobial activity of natural polysulfides found in garlic and onions.^1^ Herein, we report the antibacterial properties of several inverse vulcanized polymers against Gram-positive *Staphylococcus aureus* and Gram-negative *Pseudomonas aeruginosa*, two common causes of nosocomial infection and pathogens identified by the World Health Organization as priorities for antimicrobial development. High sulfur content polymers were synthesized with different divinyl comonomers and at different sulfur:comonomer ratios, to determine the effect of such variables on the antibacterial properties of the resulting materials. Furthermore, polymers were tested for their potential as antibacterial materials at different temperatures. It was found that the test temperature influenced the antibacterial efficacy of the polymers and could be related to the glass transition temperature of the polymer. These findings provide further understanding of the antibacterial properties of inverse vulcanized polymers and show that such polymers have the potential to be used as antibacterial surfaces.

## Introduction

Hospital-acquired infections (HAIs) are defined by the World Health Organization as infections that develop after 48 hours of hospitalization and that were not present or incubating at the time of admission.^2^ HAIs result in increased morbidity and mortality rates and financial and clinical burden for healthcare systems. The impact of HAIs is widespread, and is not confined to hospital settings, as infection can spill over into the community, as has been observed for multi-drug resistant pathogens including methicillin-resistant *Staphylococcus aureus* (*S. aureus*).^3,4^ Antimicrobial resistance (AMR) is amongst the most serious health threats of the century, and thus methods for preventing, slowing or reversing AMR are of utmost importance.^5^ In recent years, there has been interest in employing antimicrobial surfaces to help limit microbial contamination. Surfaces can act as microbial reservoirs, which upon being touched can be spread to other healthcare workers or patients and in turn lead to HAIs. Any method that can reduce the prevalence or viability of microbes on a surface could help to reduce the frequency of HAIs.^6^

**Figure 2:**
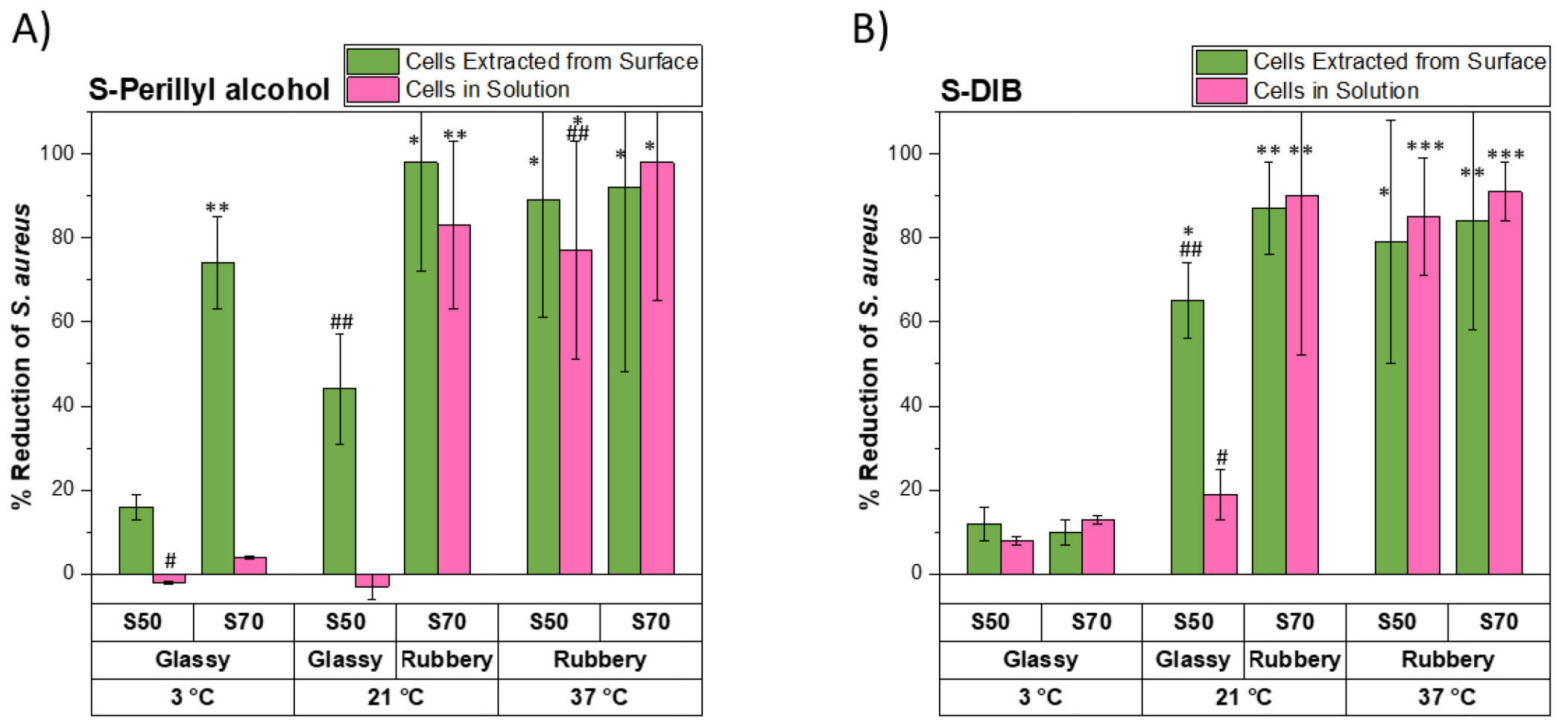
A summary of the % reduction in viable *S. aureus* cells extracted from the surface and from solution compared to polypropylene for A) S-PA and B) S-DIB at varying test temperature and sulfur:comonomer ratio. * *p*<0.05 relative to polypropylene, ** *p*<0.01 relative to polypropylene, *** *p*<0.001, # *p*<0.05 relative to polypropylene, ## *p*<0.01 for 50 wt.% sulfur compared to 70 wt.% sulfur polymers at the same test conditions.

In 2013, a new class of high sulfur content polymers coined ‘inverse vulcanized polymers’ were reported by Pyun and co-workers.^7^ Inverse vulcanization is a method for synthesizing polymers from elemental sulfur and a small amount of vinylic comonomer, the converse of conventional vulcanization which implements sulfur as a minor component of the reaction feedstock. The process consists of ring-opening polymerization of elemental sulfur followed by crosslinking with the vinylic comonomer which stabilizes the polymer from depolymerization back to elemental sulfur (Figure 1).^8^ Many vinylic comonomers have been used to synthesize inverse vulcanized polymers, such as dicyclopentadiene (DCPD),^9,10^ 1,3-diisopropenylbenzene (DIB),^7,11^ divinylbenzene (DVB),^12^ ethylidene norbornene,^13^ perillyl alcohol,^14^ and even vegetable oils such as soybean oil and sunflower oil (Figure 2).^15,16^ Properties of inverse vulcanized polymers, such as the glass transition temperature (*T*_g_) are highly dependent on the type of comonomer used and the sulfur:comonomer ratio employed in the reaction feedstock.^9^ Inverse vulcanized polymers have potential to be made inexpensively at a large scale, and have many promising applications such as improved cathode materials for Li-S batteries,^17,18^ novel optical devices,^12,19^ smart fertilisers,^16,20^ and environmental remediation by means of mercury capture,^10,21–23^ water purification and oil spill recovery.^24^

Another potential application for inverse vulcanized polymers is antimicrobial surfaces. Elemental sulfur has been documented for its antimicrobial properties throughout history, and is used dermatologically in anti-dandruff shampoos and in acne treatment.^25^ There is now growing interest in polysulfides as novel antimicrobial agents due to the antimicrobial activity of natural polysulfides found in garlic and onions.^26,1^ The main compound present in uncrushed garlic is alliin which itself has no antimicrobial activity, but is converted to antimicrobial allicin by the enzyme alliinase. Alliinase is thought to be an important defense enzyme in garlic to protect the plant from pathogenic microbes in the soil, by converting the precursor alliin to allicin. The antimicrobial activity of allicin is of interest, as the mechanism of action is different to that of conventional antimicrobial agents, and microbial pathogens do not appear to readily develop resistance against allicin.^27,28^ Due to its instability, allicin can be converted into lipid soluble diallyl sulfides such as diallyl sulfide (DAS), diallyl disulfide (DADS) and diallyl trisulfide (DATS) and other higher order diallyl polysulfides. Several studies have reported that the diallyl sulfides show an antimicrobial effect against both Gram-positive and Gram-negative pathogens, and that the antimicrobial effect increases with an increasing number of sulfur atoms in a chain (antimicrobial effect of DATS > DADS > DAS).^1,26,29^ Ajoene, another disulfide containing compound found in garlic oil has been found to inhibit quorum sensing, an important communication system used by bacteria including *Pseudomonas aeruginosa* (*P. aeruginosa*) to achieve coordinated expression of genes involved in pathogenicity. It is thought that the disulfide bond present in ajoene is vital for its antivirulence activity.^30^ Inverse vulcanized polymers are also expected to have chains of sulfur of varying lengths. In 2017, a study by Deng *et al*. found that thin layers of poly(sulfur-co-1,3-diisopropenyl benzene) (S-DIB) spin-coated onto silicon substrates were able to kill *Escherichia coli* (*E. coli*). Several S-DIB polymers with different ratios of sulfur:DIB (between 50-70 wt% sulfur) were prepared and it was found that there were no significant differences in the antibacterial activity of each polymer.^11^ In 2020 Smith *et al*. aimed to further elucidate the antibacterial properties of inverse vulcanized polymers by studying the antibacterial properties of S-DIB and S-DCPD, both synthesized with 50 wt% sulfur, against *S. aureus* and *E. coli*. The study found that S-DIB showed increased antibacterial activity against both species compared to S-DCPD at the same sulfur:comonomer ratio, and it was suggested that this could be attributed to the higher sulfur rank (length of polysulfide segments -S-(S)n-S-) of S-DIB compared to S-DCPD.^31^ It is thought that polysulfides can react with thiol-containing cellular proteins, and thus higher sulfur ranks are expected to give more labile central S-S bonds, which could react with thiol groups of bacterial proteins.^32,33^ Both studies by Deng *et al*. and Smith *et al*. show promising results for the use of inverse vulcanized polymers as antibacterial surfaces, however, further investigation is warrantedto understand in detail these properties.

Here, we report the findings of a detailed study of the antimicrobial properties of inverse vulcanized polymers. Polymers synthesized with 6 different comonomers were compared to assess the effect of the crosslinker. In addition, different sulfur:comonomer ratios were employed to evaluate the effect of the sulfur content in the polymers on their antibacterial properties. Furthermore, polymers were tested at different temperatures to assess if the antibacterial properties of the polymers differ above and below their *T*_g_. Polymers were tested against Gram-positive *S. aureus* and Gram-negative *P. aeruginosa*. Both bacterial species are problematic nosocomial pathogens, as well as causing chronic infection of the lung in people with cystic fibrosis (CF) and non-CF bronchiectasis.^34^ The *S. aureus* and *P. aeruginosa* biofilm mode of growth contributes to both prolonged survival on surfaces within the hospital environment and to the establishment and progression of infections. Furthermore, infections can often be life threatening and difficult to treat due to the emergence of multi-drug resistance strains.^35^

## Experimental

### Materials

Ground sulfur sublimed powder reagent grade ≥99.5 % was obtained from Brenntag UK & Ireland. Dicyclopentadiene (stabilized with BHT) >97%, and 1,3-Diisopropenylbenzene (stabilized with TBC) >97% were obtained from Tokyo Chemicals Industry. Divinylbenzene technical grade 80 %, (S)-(−)-Perillyl alcohol food grade ≥95%, Linseed oil, Luria–Bertani broth (Miller), LB agar, phosphate buffered saline (PBS) and glutardialdehyde were purchased from Sigma-Aldrich. Rapeseed Oil (Crisp ‘n Dry®) was purchased from Tesco. Methicillin-resistant *S. aureus* strain USA300 and *P. aeruginosa* PAO1^36^ were cultured from frozen stocks stored at the University of Liverpool.

### Methods

Polymer Synthesis: Polymerization were carried out in 40 mL glass vials placed in aluminium heating blocks. Sulfur:crosslinker weight ratios were varied between 30-70 wt% sulfur, with the total reaction scale maintained at 10 g. All reactions were begun by allowing the sulfur to melt at 135 °C, before adding the organic crosslinker under stirring. The reaction temperature was increased to 175 °C for perillyl alcohol, DIB, rapeseed oil and linseed oil, 160 °C for DCPD and maintained at 135 °C for DVB. Molded objects were prepared by transferring the reaction into a silicone mould (1 cm^3^ cubes) and curing overnight in an oven at 140 °C. The mixture was transferred from the stirred vial to the mould when the reaction mixture had become homogeneous and viscous (an aliquot of the reaction mixture, when removed on a spatula and allowed to cool to room temperature, would no longer visibly separate to clear organic monomer, and precipitated yellow sulfur powder).

The loss of peaks corresponding to alkene units in the Fourier-transform infrared spectroscopy were monitored to confirm consumption of the comonomers during the reaction (Figure S1-S6). ^1^H Nuclear magnetic resonance (NMR) spectra were also obtained for polymers with a soluble fraction (Figure S7-10) to assess the consumption of alkene units.

Bacteria Preparation, Storage and Enumeration: Glycerol stocks of *Staphylococcus aureus* (*S. aureus*) strain USA300 and *Pseudomonas aeruginosa* (*P. aeruginosa*) strain PAO1 were stored at -80 °C for long term storage. For experimental use, frozen glycerol stocks of *S. aureus* and *P. aeruginosa* were defrosted and spread onto LB agar plates which were incubated overnight at 37 °C. Bacterial cultures were prepared by swabbing one colony into 10 ml LB broth followed by overnight incubation at 37 °C. Colony forming units (CFU) were enumerated by serially diluting the cultures in PBS onto LB agar, using the Miles and Misra method. CFU/cm^2^ and CFU/ml were calculated using the following equation:

CFU=(no.ofcoloniesxdilutionfactor)/volumeofcultureplate

Viable Cell Enumeration Assay: *S. aureus* USA300 and *P. aeruginosa* PAO1 were used to evaluate the antibacterial efficiency of the sulfur polymers molded into 1 cm^3^ cubes using polypropylene cubes as a negative control. 1 cm^3^ cubic samples were soaked in ethanol, allowed to air dry and placed in separate wells of a 24-well plate. Overnight cultured bacteria prepared in LB broth were diluted to 10^5^ CFU/mL (OD600 = 0.001). 1.2 mL of diluted bacterial solution was added to each sample in the 24-well plate and allowed to incubate statically. For *S. aureus*, incubation times were 5 h at 37 °C and 24 h at room temperature. For *P. aeruginosa*, the incubation time was 3 h at 37 °C. After incubation, the bacterial solution surrounding each sample was removed and the viable cells were enumerated after serial dilution of the solution in PBS onto LB agar, using the Miles and Misra method. The cubic samples were gently rinsed with 1 mL of PBS to remove any planktonic cells and were vortexed at high speed in 1 mL of LB broth for 10 s to remove any adhered cells. The vortexed solution was serially diluted in PBS and the viable cells associated with the sample surface were enumerated using the Miles and Misra method on LB agar, as above. All samples were tested in technical triplicate.

Statistical analysis: Statistical analysis was conducted using one-way analysis of variance (ANOVA) on the calculated CFU/cm^2^ values for the sample surface and the calculated CFU/ml in solution. Differences were deemed as statistically significant if a value of *p* < 0.05 was obtained.

Biofilm Staining Assay: 1 cm^3^ samples (high sulfur content polymers and polypropylene) were placed in separate wells of a 24-well plate. Overnight cultured bacteria prepared in LB broth was diluted to 10^5^ CFU/mL (OD600 = 0.001). 1.2 mL of diluted bacterial solution was added to each sample well and allowed to incubate statically for 5, 24 and 48 h at 37 °C. After static incubation the samples were washed with 1.2 mL PBS and allowed to dry in a new 24-well plate. Once dried, 1.2 mL of 0.25% crystal violet stain was added to each well containing samples and was kept for a minimum of 30 minutes at room temperature. The samples were washed thoroughly with water to remove excess stain and were allowed to dry. 1.2 mL of ethanol was used to solubilize the remaining dye on each sample. The absorbance of the solubilized dye was measured at 600 nm using ethanol as a blank.

Leaching Study: 1 cm^3^ cubic samples were placed in separate wells of a 24-well plate. To each sample, 1.2 mL of distilled water was added. The well plate was incubated at 37 °C for 24 h. The solution surrounding the samples was removed and analyzed by inductively coupled plasma optical emission spectrometry (ICP-OES).

### Characterization

Differential Scanning Calorimetry (DSC): DSC was performed using a TA Instruments Q200 DSC, programmed using a heat/cool/heat method for 3 cycles by heating to 150 °C, cooling to -80 °C and re-ramping to 150 °C. The heating/cooling rate was set to 10 °C per minute. The second heating curve was analyzed and used to determine the glass transition temperature.

Water Contact Angle Measurements (WCA): Static WCA measurements were obtained using a DSA100 Expert Drop Shape Analyzer using the sessile drop mode and Young-Laplace fitting method. 5 μL water droplets were used and a minimum of 3 measurements were recorded for each material analyzed.

Scanning electron microscopy (SEM) and energy-dispersive x-ray spectroscopy (EDS): SEM and EDS was performed using a Hitachi S-4800 cold-field emission scanning electron microscope. 1 cm^3^ polymer samples were mounted onto SEM stubs using conductive silver paint. Prior to imaging, samples were coated with gold using a current of 120 mA for 15 s to give approximately 15 nm gold coatings using a Quorum S1505 ES sputter coater. For imaging bacterial cells, the samples were incubated for 2 h with *S. aureus* USA300. Following incubation, the samples were fixed with 2.5% glutardialdehyde solution in sterile PBS for 4 h at 4 °C and were dehydrated with increasing concentrations of ethanol (30, 50, 75, 90, 95, and 100% v/v) for 10 min in each concentration. After drying, the samples were mounted onto SEM stubs and coated as above.

## Results and discussion

### Synthesis and Characterization

Inverse vulcanized polymers were synthesized by adding the vinylic comonomer to molten sulfur at 135 °C and further heating between 135 °C and 175 °C, depending on the comonomer used, until the mixture became homogeneous and viscous. The reaction mixture was poured into 1 cm^3^ cube shaped silicone moulds and transferred to an oven to be cured overnight at 140 °C. Following curing, the polymers molded into 1 cm^3^ cubes were removed from the silicone moulds and used for antibacterial testing. ^1^H NMR and FT-IR spectroscopy were used to compare the alkene units present in the unreacted comonomers to the resultant polymers. Loss of peaks corresponding to alkene units suggests successful reaction between sulfur and the comonomers. The *T_g_* of the resultant materials was determined by DSC. DSC was also used to determine the presence of any crystalline sulfur within the materials, the presence of which would suggest that unreacted elemental sulfur remains within the polymer.

Several polymers were synthesized (denoted S-comonomer) with different comonomers, sulfur:comonomer ratios and thus different *T*_g_ (See Table 1), to further understand the antibacterial properties of inverse vulcanized polymers. As the temperature rises above the *T*_g_, amorphous polymers undergo a phase change from a glassy, rigid state to a rubbery, flexible state.^37^ Three sets of materials differing by their *T*_g_ range were chosen to assess whether antibacterial activity was isolated to individual comonomers or if similar antibacterial activity was seen within a given set, and therefore dependent upon the *T*_g_. The first set of comonomers chosen included DCPD and DVB, as they result in insoluble crosslinked polymers with high *T*_g_ far exceeding room temperature (>45 °C) when reacted with elemental sulfur. The second set of polymers synthesized used DIB and perillyl alcohol as comonomers, their respective sulfur polymers result in polymers with lower *T*_g_ (0-40 °C). In addition to this they are not fully crosslinked polymers as they are soluble in organic solvents such as chloroform and THF. The third set of polymers were synthesized from vegetable oils, namely linseed oil and rapeseed oil. Inverse vulcanized polymers synthesized with vegetable oils have lower *T*_g_ (<0 °C) due to the flexibility of long carbon chains present in triglycerides.

The sulfur rank (length of polysulfide segments -S-(S)n-S-) of inverse vulcanized polymers is expected to be dependent on both the comonomer and the sulfur:comonomer ratio employed in the reaction feedstock. Increasing the amount of sulfur in the feedstock is expected to give higher sulfur ranks, however, this also depends on the ability of the comonomer to stabilize large amounts of sulfur. There is a limit to the maximum sulfur rank that can be achieved. Increasing the sulfur content beyond the limit at which it can be stabilized by the comonomer will no longer give higher sulfur rank, and instead the material becomes a composite that consists of polymer and elemental sulfur.^14^ Determination of the sulfur rank of inverse vulcanized polymers by analytical methods remains challenging. However, unreacted crystalline S_8_ that is not incorporated into the polymer chains and therefore cannot contribute to the sulfur rank can be detected by methods such as DSC. The theoretical average sulfur rank in the polymers can be calculated, providing that all S8 was consumed during reaction (determined by a lack of a sulfur melting transition in the DSC trace) and by calculating the molar ratio of alkene units to sulfur atoms.^15^ The average sulfur rank for the polymers synthesized for this study is shown in Table 1. The polymers are likely to have various sulfur ranks within the chains, however, the calculated average sulfur rank provides a useful comparative value. The average sulfur rank for S-LO and S-RO could not be calculated, as the molar ratio of alkene units is unknown due to the oils being composed of mixed triglycerides.

### Antibacterial Properties

Antibacterial testing of all polymers was carried out by submerging the polymer cubes (1 cm^3^) in bacterial culture, followed by incubation. Polypropylene cut to 1 cm^3^ cubes was used as a negative control for all tests, and each sample was tested in triplicate. Three incubation temperatures were used with the aim of testing the samples at their glassy state (T < *T*_g_) and at their rubbery state (T > *T*_g_). The three temperatures chosen were (3 ± 1) °C, (21 ± 1) °C and (37 ± 0.3) °C. The reduction in viable cells in solution and viable cells extracted from the surface of the materials was investigated. To enumerate the viable cells in solution, after incubation, the bacterial solution surrounding each sample was removed and the viable cells were enumerated after serial dilution of the solution in PBS onto LB agar, using the Miles and Misra method.^38^ To enumerate the viable cells extracted from the surface of the materials, the cubic polymers were gently rinsed with PBS to remove any planktonic cells and were vortexed in LB broth to remove any adhered cells. The vortexed solution was serially diluted in PBS and the viable cells associated with the sample surface were enumerated using the Miles and Misra method.

S50-PA and S70-PA were found to have *T*_g_ of (34 ± 3) °C (Figure S11) and (14 ± 4) °C (Figure S12) respectively. S50-PA and S70-PA were tested against a methicillin-resistant *S. aureus* strain (USA300) at (3 ± 1) °C, (21 ± 1) °C and (37 ± 0.3) °C. The polymers behaved differently at each temperature (Figure 3a). At 3 °C, neither of the polymers showed a reduction in viable cells in solution compared to polypropylene, and only S70-PA showed a reduction in surface associated cells (74 % reduction). At this temperature, both polymers are in their glassy state (T < *T*_g_). It is possible that S70-PA has greater antibacterial activity at this temperature compared to S50-PA due to higher sulfur content and thus a higher sulfur rank (length of polysulfide segments -S-(S)n-S-) and more labile S-S bond that could react with thiol containing proteins of the bacteria.^32,33^ By increasing the test temperature to 21 °C, S50-PA showed a moderate reduction in surface associated viable cells (44 % reduction relative to polypropylene control, *p*=0.06), where at lower test temperature no effect was seen, however, no reduction in viable cells in solution was observed. For S70-PA at 21 °C, the reduction in surface associated cells appears similar to the result at 3 °C, however, there is a reduction in the viable cells in solution (83 % reduction). At 21°C, S50-PA is in its glassy state (T < *T*_g_), whereas S70-PA is in its rubbery state (T > *T*_g_). When the test temperature was increased further to 37 °C both S50-PA and S70-PA show a reduction in the viable cells extracted from the surface (89% for S-50PA and 92% for S70-PA) and from solution (77% for S50-PA and 98% for S70-PA). It is worth noting that S70-PA had lost its shape and yellow precipitates were present on the materials surface after incubation at 37 °C. The results show that both S50-PA and S70-PA show an antibacterial effect against *S. aureus*, however, this effect is dependent on the test temperature and could therefore be related to the physical state of the polymer. Little antibacterial effect is seen when the test temperature is well below the *T*_g_ of the polymers, however, when the temperature approaches the *T*_g_ of the polymers, there is a reduction in surface associated viable cells. A reduction in the viable cells in solution is seen when the test temperature is above the *T*_g_ of the polymers.

To assess if the observation that the antimicrobial effect is dependent on the physical state of the polymer (i.e rubbery or glassy) is limited to individual comonomers, or, if it is seen for other polymers with similar *T*_g_s synthesized with different comonomers, S-DIB was also studied. S50-DIB and S70-DIB gave *T*_g_s of (27 ± 1) °C (Figure S13) and (10 ± 4) °C (Figure S14), therefore at each test temperature the state of each sample should be the same as for the perillyl alcohol equivalents (at 3 °C both samples are glassy, at 21 °C 50 wt% sulfur is glassy and 70 wt% sulfur is rubbery, at 37 °C both samples are rubbery). The results of the percentage reduction in viable surface associated cells and the viable cells in solution for S-DIB are shown in Figure 3b. The results show a similar trend to that of the perillyl alcohol equivalents, whereby when the polymers are in their glassy state at a test temperature of 3 °C, no significant antibacterial effect is seen against *S. aureus* for either cells in solution or surface associated cells. At 21 °C both polymers show a reduction in surface associated viable cells, but only S70-DIB shows a significant reduction in the viable cells in solution, and at 37 °C both polymers show a reduction in both the surface associated cells (79% for S50-DIB and 84% for S70-DIB) and the cells in solution (85% for S50-DIB and 91% for S70-DIB).

The tests against *S. aureus* were also repeated for the other two sets of polymers, the high *T*_g_ set synthesized from DCPD and DVB, and the low *T*_g_ set synthesized from linseed oil and rapeseed oil (Table 2). Due to their high *T*_g_ (45-88 °C) (Figures S15-S18), the polymers synthesized from DCPD and DVB were tested only at 37 °C, as the polymers will be in the glassy state at all test temperatures. The results show that S50-DCPD and S50-DVB show no, or very little reduction in the viable surface associated cells and viable cells in solution compared to polypropylene. S70-DCPD and S70-DVB show a greater reduction in viable surface associated cells compared to the 50 wt.% sulfur equivalents, suggesting that the sulfur content of the polymers is influencing their antibacterial properties. These results are also consistent with the observations made by Smith *et al*. whereby S-DCPD synthesized at 50 wt.% sulfur did not show an antibacterial effect against *S. aureus* whereas S-DIB did show an inhibitory effect.^31^ It was suggested that these differences could be due to the higher sulfur rank expected for S-DIB compared to S-DCPD, which could explain why S70-DCPD in this study does show a moderate reduction in the viable surface associated cells compared to polypropylene whereas S50-DCPD does not show an inhibitory effect. S-polymers using rapeseed and linseed oil as comonomers were synthesized at 30 and 50 wt.% sulfur, due to such vegetable oils only being able to stabilize lower amounts of sulfur.^14^ Both polymers show signs of inhomogeneity, especially at 50 wt.% sulfur feeds whereby crystalline sulfur was visible on the sample surface. The polymers formed from rapeseed and linseed oil at 30 and 50 wt.% sulfur have *T*_g_s below 0 °C (Figures S19-S22), and therefore these samples were tested against *S. aureus* at 3 °C, a temperature at which the polymers exhibit their rubbery state. S30-RO, S50-RO, S30-LO and S50-LO sulfur show a reduction in viable surface associated cells and viable cells in solution.

The polymers from all six of the comonomers tested against *S. aureus* showed a correlation between the % reduction in viable cells in solution and whether the sample was in the rubbery or glassy state in the testing temperature. All samples tested above their *T*_g_ showed a greater than 25% reduction, whereas all samples tested below their *T*_g_ showed a less than 25% reduction (see Figure S23). A possible reason for an enhanced % reduction in viable cells in solution when the polymers are tested above their *T*_g_ could be the leaching of antimicrobial species from the polymer. Above the *T*_g_, when the polymer is in its rubbery state, polymer chains have increased mobility and could allow for the diffusion of any potential antimicrobial species from the polymer to the surface, from where they could then leach into solution.

High sulfur content terpolymers (ternary copolymers) using perillyl alcohol and DCPD were synthesized and tested against *S. aureus* in order to investigate the antibacterial activity with increasing wt.% of DCPD. The terpolymers, denoted S-PA-DCPD, were synthesized at 50 wt.% sulfur and varying the PA:DCPD ratio (Table S1) by increasing the DCPD content in 5 wt.% increments. The *T*_g_ of the resultant materials increased, whilst the soluble fraction of the materials decreased with increasing wt.% DCPD (Figure S24). The increase in *T*_g_ and decrease in soluble fraction of the terpolymers with increasing wt.% DCPD can be attributed to higher crosslink density provided by DCPD. Terpolymers synthesized with S:PA:DCPD ratios of 50:45:5, 50:35:15 and 50:25:25 were tested against *S. aureus* and compared to S50-PA and S50-DCPD copolymers (Figure S25). The results show that the terpolymers have different antibacterial properties depending on the ratio of PA:DCPD employed. The S-PA-DCPD terpolymer with a ratio of 50:45:5 showed a similar reduction in the viable cells on the surface, relative to polypropylene, to that of S50-PA. However, the solution effect of the 50:45:5 terpolymer is diminished compared to S50-PA. The *T*_g_ of S50-PA is 34 °C, but doping the polymer with 5 wt.% DCPD increased the *T*_g_ to 41 °C. At the test temperature of 37 °C, S50-PA is rubbery whereas the 50:45:5 terpolymer is glassy. It is possible that the solution effect is decreased due to the increase in *T*_g_ resulting in lower mobility of the polymer chains for the 50:45:5 terpolymer, which could prevent any potential antimicrobial species from leaching out into solution. As the DCPD content is increased further to 15 and 25 wt.%, there is a decrease in the reduction of viable cells on the surface compared to S50-PA and the 50:45:5 terpolymer. The decrease in surface effect could be attributed to a decrease in the average sulfur rank with increasing DCPD content (Table S1).

**Figure 4:**
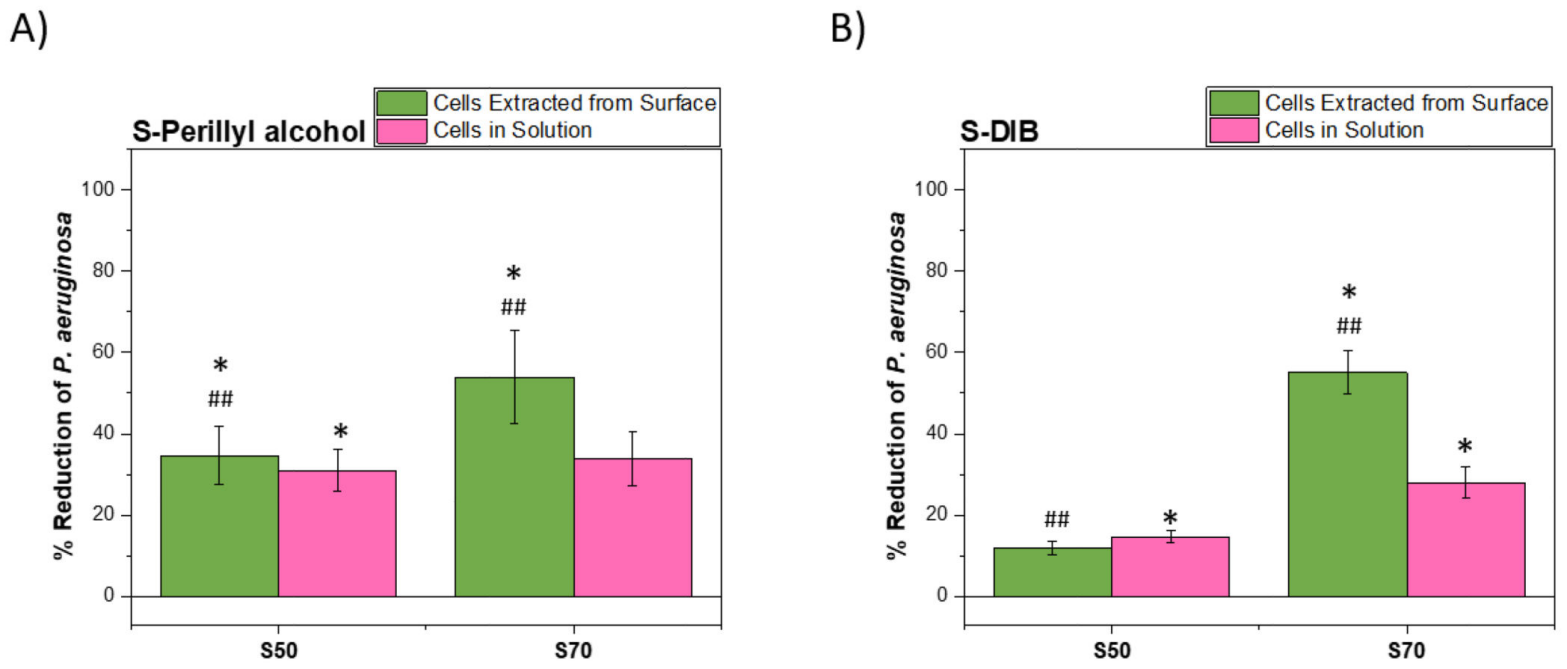
A summary of the % reduction in viable *P. aeruginosa* cells extracted from the surface and from solution compared to polypropylene for A) S50-PA and S70-PA and B) S50-DIB and S70-DIB. *p<0.05 relative to polypropylene ##*p*<0.01 for 50 wt% sulfur compared to 70 wt% sulfur polymer.

*P. aeruginosa* is a Gram-negative, rod-shaped pathogen that is a leading cause of persistent nosocomial infections.^38^ S50-PA, S70-PA, S50-DIB and S70-DIB were tested against *P. aeruginosa* at 37 °C. The polymers drove a reduction in the viable cells both in solution and on the polymer surface (Figure 4). ANOVA analysis demonstrates that S50-PA and S70-PA show a similar reduction in the viable cells in the solution that surrounded the samples during incubation, however, S70-PA shows a greater reduction in the viable cells associated with the surface (p<0.01 vs S50-PA). For the S-DIB equivalents against *P. aeruginosa*, S70-DIB results in a greater reduction in both the viable cells in solution and on the surface compared to S50-DIB, and the results are comparable to that of S70-PA.

**Figure 5:**
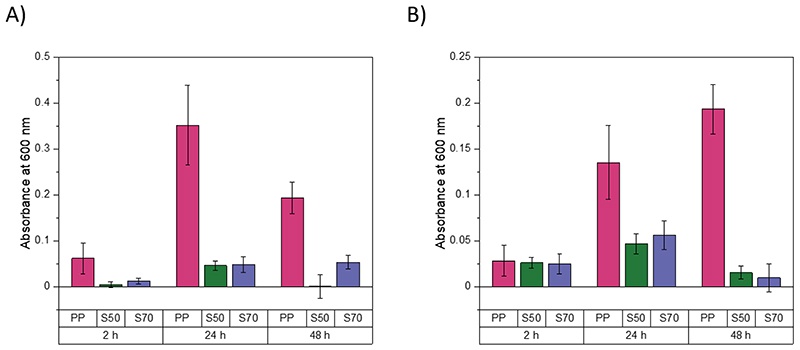
Absorbance at 600 nm for A) S-PA and B) S-DCPD, after staining with crystal violet at 2, 24 and 48 h incubation times with *S. aureus*. Where PP: polypropylene, S50: 50 wt.% sulfur polymer and S70: 70 wt.% sulfur polymer.

Pathogens that can form biofilms are less sensitive to antibiotic treatment than planktonic cells.^39^ *S. aureus* and *P. aeruginosa* can both form biofilms, both on environmental surfaces and during infection.^40^ Therefore the development of antibacterial surfaces that can prevent bacterial adhesion and reduce the ability of pathogens to form biofilms are of particular interest.^41^ A methicillin-resistant *S. aureus* strain (USA300) was used to investigate the ability of high sulfur content polymers to inhibit biofilm formation on their surfaces. Briefly, cubic polymers were incubated statically with *S. aureus* at 37 °C for 2, 24 and 48 h to allow for biofilm growth. After incubation, the samples were rinsed with PBS and stained with 0.25 % crystal violet stain. Excess stain was washed off thoroughly with water. After washing, the stain was solubilized with 1.2 ml ethanol and the absorbance of the solution was measured at 600 nm. The absorbance at 600 nm is related to the amount of biofilm on the sample surface, which becomes stained with crystal violet during the staining process. At 2, 24 and 48 h incubation periods at 37 °C, S50-PA and S70-PA have lower absorbance values at 600 nm compared to polypropylene (Figure 5a), which suggests that biofilms form more readily on the surfaces of polypropylene. The study was also repeated at 3 and 21 °C to cover all temperatures at which S-PA was tested for its antibacterial activity (Figure S26 and S27). The absorbance at 600 nm after 24 h at 3 °C was similar for polypropylene, S50-PA and S70-PA. After 48 h incubation, the absorbance at 600 nm was higher for polypropylene compared to both S-PA samples, suggestive of lower biofilm prevalence on S50-PA and S70-PA compared to polypropylene. The absorbance at 600 nm after 24 and 48 h incubation for polypropylene at 3 °C was lower than that at 37 °C, as expected due to slower growth and biofilm formation by bacteria at lower temperatures. At incubation temperatures of 21 °C, at both 24 and 48 h (Figure S27) the absorbance at 600 nm was highest for polypropylene and lowest for S70-PA. The absorbance values are also consistent with the results shown in Figure 3A, whereby less viable cells were extracted from the surface of S70-PA relative to S50-PA. *P. aeruginosa* biofilm formation was also studied on the surfaces of S-PA (Figure S28) and it was found that the absorbance at 600 nm for S50-PA and S70-PA was reduced by more than half compared to the absorbance for polypropylene. *S. aureus* biofilm staining results for S-DIB after 24 and 48 h incubation at 37 °C (Figure S29) suggest lower biofilm prevalence on the surfaces of S-DIB compared to polypropylene. The absorbance values at 600 nm are similar for both S50-DIB and S70-DIB, consistent with the results shown in Figure 3B, whereby both polymers showed a similar % reduction in viable *S. aureus* cells extracted from the surface. S50-DCPD and S70-DCPD were tested against *S. aureus*. After 2 h incubation, the absorbance value for S50-DCPD and S70-DCPD are similar to that of polypropylene (Figure 5b), which suggests that there is a similar amount of biofilms on the surfaces of the samples. However, after 24 and 48 h incubation periods, the absorbance values for both S50-DCPD and S70-DCPD are lower than that of polypropylene, suggestive of lower biofilm prevalence on the S-DCPD samples compared to polypropylene. *P. aeruginosa* biofilm formation is also inhibited by S-DCPD, relative to polypropylene, as shown in Figure S30, whereby the absorbance at 600 nm was reduced by more than half for S50-DCPD and S70-DCPD in comparison to polypropylene (Figure S31 and S32). Biofilm formation on the surfaces of S-DVB was also investigated, and it was found that the absorbance at 600 nm after 24 and 48 h incubation with *S. aureus* at 37 °C was lower for S50-DVB and S70-DVB compared to polypropylene (Figure S33). These results suggest that high sulfur content polymers can inhibit biofilm formation on their surfaces. *S. aureus* biofilm formation on the surfaces of S30-LO and S50-LO was investigated at an incubation temperature of 37 °C (Figure S34). After 24 and 48 h incubation the absorbance values for polypropylene were higher than S30-LO and S50-LO. The antibacterial activity for both S30-LO and S50-LO was tested at 3 °C (Table 2), at this temperature both polymers show similar activity relative to polypropylene, however, the biofilm staining results at 37 °C suggest that *S. aureus* biofilm growth is inhibited on the surface of S50-LO to a greater extent than for S30-LO. *S. aureus* biofilm formation on the surfaces of S-RO was studied at incubation times of 24 and 48 h at 37 °C (Figure S35). After 24 h and 48 h it was found that the absorbance values at 600 nm were greater for polypropylene compared to S30-RO and S50-RO. After 48 h incubation with *S. aureus*, the absorbance values at 600 nm were similar for S30-RO and S50-RO, consistent with the results in Table 2, whereby the % reduction in viable *S. aureus* cells extracted from the surfaces was also similar for both samples.

### Surface Wettability and Topography

**Figure 6:**
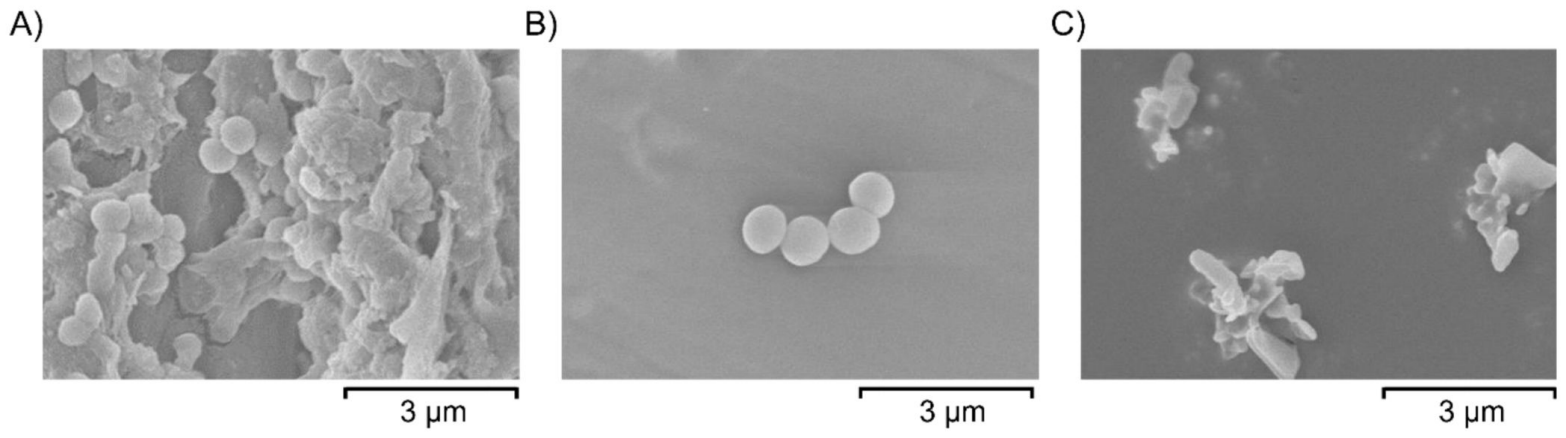
SEM images of A) polypropylene B) S50-DCPD and C) S50-PA after incubation with *S. aureus*.

To investigate if the variation in antibacterial activity with different comonomers is due to differences in the sample surfaces, the wettability and surface morphologies were assessed. The wettability of the surfaces was measured by dropping 5 μL droplets of water onto the surfaces and measuring the contact angle formed using the Young-Laplace fitting method. All polymers tested have water contact angles >90 ° and are therefore hydrophobic, however, no correlation was found between the antibacterial activity of the polymers and their hydrophobicity (see Figure S36). The morphology of the surfaces was visualized by scanning electron microscopy (SEM) to probe whether any differences in the roughness of the sample surface might be playing a role in the antibacterial activity. Neither S50-PA, nor S50-DCPD, have a completely smooth surface (Figure S37). Samples were also imaged after incubation with *S. aureus* to visualize the cells on the surface. Spherical species with diameters of approximately 0.5-1 μm were frequently observed on the surface of polypropylene, which were not visible on the surface of clean polypropylene (Figure S38). The size and shape of these species are consistent with the appearance of *S. aureus* cells, which appear to be growing within a biofilm, with visible extracellular matrix structures.^42^ *S. aureus* cells were also found on the surface of S50-DCPD, albeit at reduced abundance relative to the polypropylene sample, however, no biofilms were seen. No biofilms or cells were found on the surface of S50-PA (Figure 6), as suggested from the biofilm staining assay using crystal violet (Figure 5). The non-spherical species visible on the surface of S50-PA after incubation with *S. aureus* (Figure 6) are also visible on the images of clean S50-PA (Figure S37). These non-spherical species could be crystalline sulfur.

The chemical composition of the surfaces was also compared by Energy-dispersive X-ray spectroscopy (EDS) (Figure S37) which shows a similar distribution of both sulfur and carbon on the surfaces of both S50-PA and S50-DCPD. Oxygen was detected in the EDS spectrum of S50-PA, which could be due to the alcohol group on perillyl alcohol.

### Polymer Leaching Study

To further understand what is causing the reduction in viable cells in solution, a leaching study was conducted. The observation that some of the samples result in a reduction in viable cells in the solution that surrounds the samples suggests that active antibacterial species may be leaching out of the polymers.^43^ The reduction in *S. aureus cells* in the presence of S50-PA and S70-PA in solution showed time-dependency when tested at 21 °C (Figure S39). No significant reduction in *S. aureus* cells in solution was observed after 5 h for both polymers relative to polypropylene, and, after 24 h only S70-PA showed a reduction relative to polypropylene. However, after 48 h, S50-PA and S70-PA show a reduction in viable *S. aureus* cells in solution compared to polypropylene. At 21 °C the S50-PA is glassy whereas the S70-PA is rubbery. It is expected that antibacterial species that may be leaching out of the polymers should do so at a faster rate for a rubbery polymer, where the mobility of the polymer chains aids the migration of active species to the surface of the sample.^44^ In order to investigate what species may be leaching out of the sulfur polymers, S70-PA was studied. Briefly, 1 cm^3^ cubic samples of S70-PA were incubated at 37 °C in 1.2 mL deionized water (or D2O for NMR analysis) for 24 h. After incubation, the samples had lost their shape and yellow precipitates were present on the surface (see Figure S40). DSC analysis was conducted on the samples before and after incubation, which suggests that the yellow precipitate is elemental sulfur due to the presence of a sulfur melting peak in the thermogram after incubation where it was not present in the thermogram prior to incubation (Figure S12 and S41). The D2O solution which surrounded the samples was subjected to ^1^H NMR analysis to detect any unreacted perillyl alcohol or any oligomers that were potentially leaching out of the samples, no peaks were present in the spectra, which suggests that no species containing hydrogen were detectable in the leachate. The solution in water was also submitted for Inductively Coupled Plasma Optical Emission Spectroscopy (ICP-OES) analysis to determine if any sulfur species were leaching out into solution, and it was found that sulfur was detectable in the leachate at a concentration of 2 ppm. As yellow precipitates were visible on the sample surfaces after incubation, and as sulfur was detected in the leachate by ICP-OES, it is possible that unreacted sulfur migrates to the surface when the polymer is held at a temperature above its *T*_g_ and could therefore leach out of the sample into the surrounding medium. It is also worth noting that although no crystalline elemental sulfur was detected by DSC prior to incubation, thin-layer chromatography analysis of the sample suggested the presence of elemental sulfur (Figure S42). There are several possible antibacterial species that could be leaching out of the polymers such as elemental sulfur, unreacted comonomers, and hydrogen sulfide. Further studies will be required to investigate what species can leach out of the polymers and if such species are antibacterial. Antibacterial materials that rely on the leaching of bactericidal species are known to have finite antibacterial activity, therefore the longevity of the solution effect will also require further investigation.^45^ The mode of action of inverse vulcanized polymers against bacteria is still unclear, however, this study shows that the polymers have an inhibitory effect against Gram-positive and Gram-negative bacterial cells both on the surface and in solution, and that the effect is related to the *T*_g_ of the materials.

## Conclusions

In summary we have shown that inverse vulcanized polymers show an inhibitory effect against Gram-positive *S. aureus* and Gram-negative *P. aeruginosa*, and that the antibacterial efficacy of such polymers is dependent on the comonomer employed and the sulfur:comonomer ratio. In addition, we show that biofilm formation is inhibited on the surfaces of inverse vulcanized polymers relative to polypropylene. We demonstrate that the antibacterial activity of the polymers varies with the temperature at which they are tested, which suggests that the *T*_g_ of the polymers is an important parameter for antibacterial applications. These observations suggest that polymers with tuneable antimicrobial properties can be synthesized based on their *T*_g_, and that the properties can be switched dependent on the temperature at which they are employed. The mechanism of action of inverse vulcanized polymers against bacteria remains unclear, particularly for the surface activity. The exact nature of how sulfur containing polymers interact with bacteria is not yet fully understood. Possible routes include sulfur-sulfur bond interactions with cysteines in bacterial cell wall/outer membrane proteins or release of superoxide radical anions interfering with bacterial metalloproteins.^47^ Polysulfides are redox active, and H_2_S_x_ species can reduce dioxygen to O_2_^•−^, causing radical generation and oxidative stress.^47, 48^ Whether this action takes place by a direct interaction of the bacteria with the surface of the polymer, or by the release of a sulfide species from within the polymer, it is rational that the glass transition temperature, and therefore physical state, or the polymer would influence these processes. Below the *T*_g_, the polymer chains are held in a rigid, glassy state, with little movement possible. This would be expected to hinder not only the mobility of the chains themselves, but also the diffusion of any species into or out of the polymer structure. Above the *T*_g_, in the rubbery state the polymer chains would be free to move, and therefore with more chance of collisions of S-S bonds or radical chain ends with bacterial outer membrane or cell wall components. There would also be much more rapid diffusion out of the polymer of any low molecular weight polysulfide species (e.g. RS_x_H) generated by the redox behavior of the bacteria.

These results suggest that some inverse vulcanized polymers could be leaching potential antibacterial agents such as elemental sulfur and that the *T*_g_ is an important parameter to consider. In conclusion, inverse vulcanized polymers show potential for use antibacterial surfaces that can act against both Gram-positive and Gram-negative bacteria and can also inhibit biofilm formation.

## Supplementary Material

Supporting Information

## Figures and Tables

**Figure 1:**
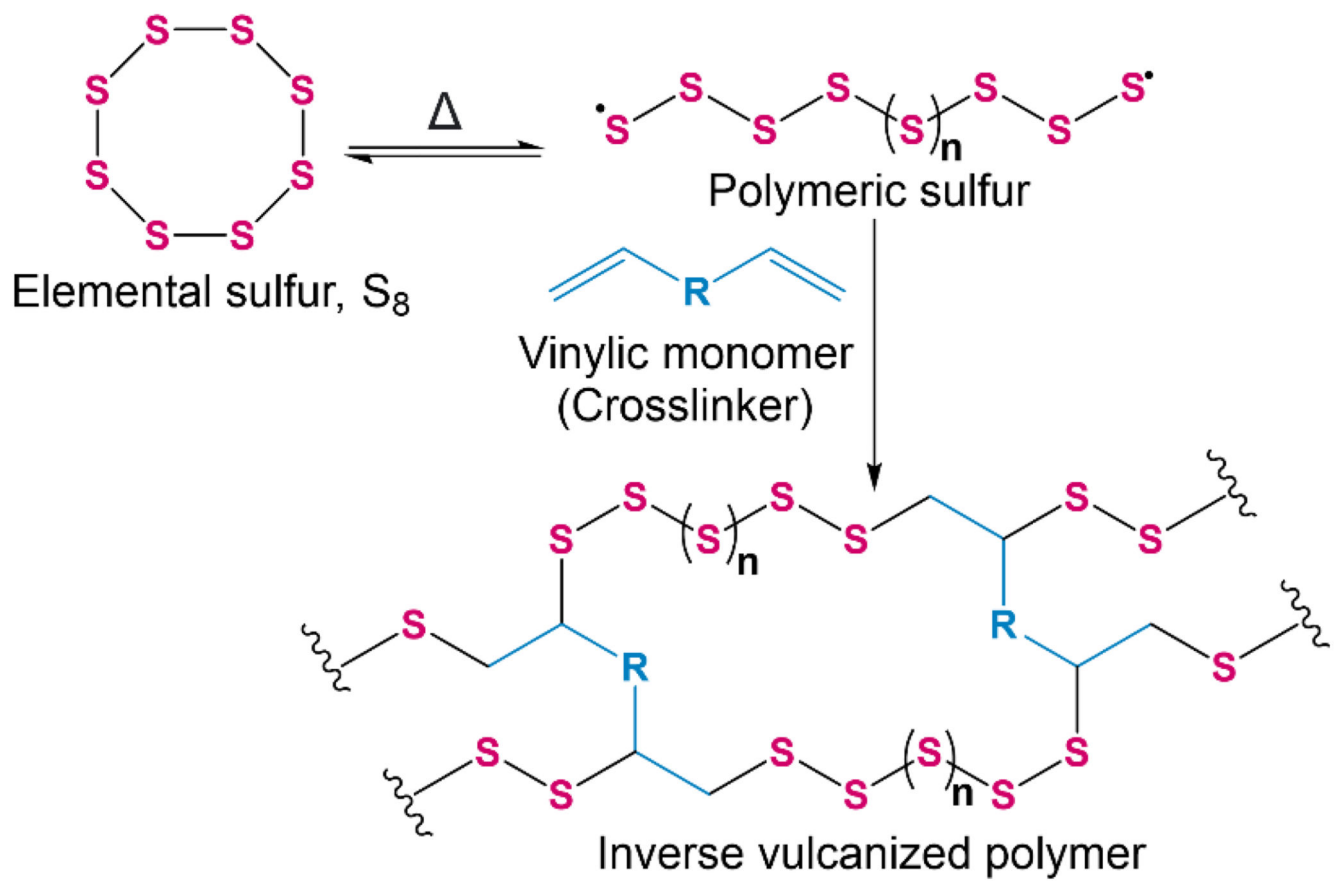
A general scheme for the inverse vulcanization of sulfur with a vinylic comonomer.

**Figure 1:**
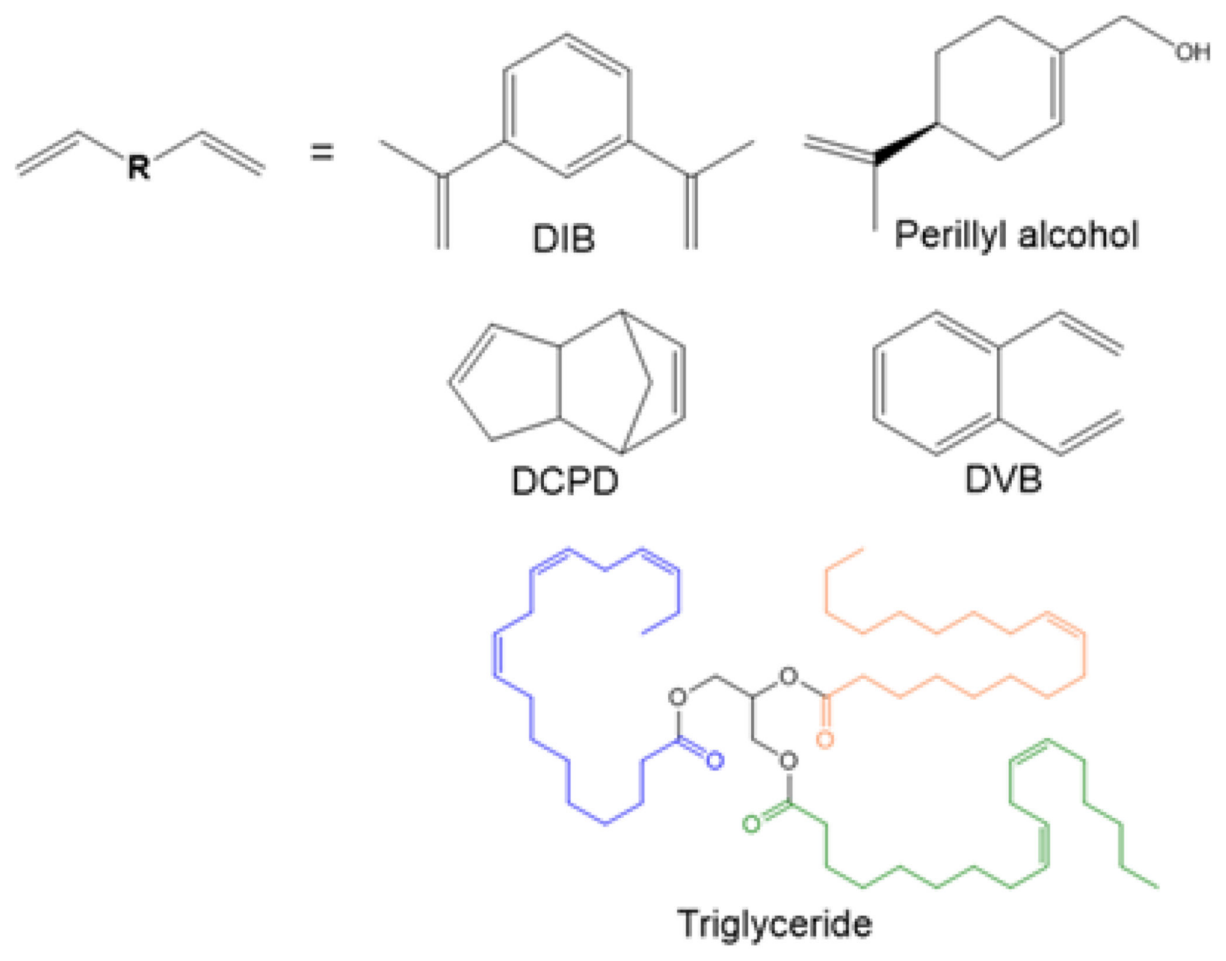
The structures of the comonomers used in the study: 1,3-diisopropenylbenzene (DIB), perillyl alcohol, dicyclopentadiene (DCPD), divinylbenzene (DVB) and an example of triglyceride which is a major component of vegetable oils such as linseed oil.

**Table 1:** A summary of the polymers synthesized, detailing the comonomer and the ratio of sulfur:comonomer employed in the reaction feedstock, the *T*_g_ of the resultant materials and their solubility in chloroform (S: soluble, SS: sparingly soluble, IS: insoluble).

Polymer	Comonomer	wt.% sulfur	Wt.% comonomer	*T_g_* (°C)	Solubility	Average sulfur rank
S50-PA	Perillyl alcohol	50	50	34	S	2.4
S70-PA	Perillyl alcohol	70	30	14	S	5.5
S50-DIB	DIB	50	50	27	s	2.5
S70-DIB	DIB	70	30	10	S	5.7
S50-DCPD	DCPD	50	50	88	IS	2.1
S70-DCPD	DCPD	70	30	45	IS	4.8
S50-DVB	DVB	50	50	72	IS	2
S70-DVB	DVB	70	30	52	IS	4.7
S30-RO	Rapeseed oil	30	70	-31	SS	-
S50-RO	Rapeseed oil	50	50	-30	SS	-
S50-LO	Linseed oil	30	70	-12	SS	-
S50-LO	Linseed oil	50	50	-12	SS	-

Polymer	Comonomer	wt.% sulfur	wt.% comonomer	*T_g_* (°C)	Solubility	Average sulfur rank*
S50-PA	Perillyl alcohol	50	50	34	S	2.4
S70-PA	Perillyl alcohol	70	30	14	S	5.5
S50-DIB	DIB	50	50	27	S	2.5
S70-DIB	DIB	70	30	10	S	5.7
S50-DCPD	DCPD	50	50	88	IS	2.1
S70-DCPD	DCPD	70	30	45	IS	4.8
S50-DVB	DVB	50	50	72	IS	2
S70-DVB	DVB	70	30	52	IS	4.7
S30-RO	Rapeseed oil	30	70	-31	SS	-
S50-RO	Rapeseed oil	50	50	-30	SS	-
S30-LO	Linseed oil	30	70	-12	SS	-
S50-LO	Linseed oil	50	50	-12	SS	-

* Theoretical average sulfur rankgiven by: (molesof S stoms / molesof alkene units)

**Table 2:** A summary of the % reduction in viable *S. aureus* cells extracted from the surface and from solution compared to polypropylene for various S-polymers. The *T*_g_ and test temperature are also summarised for each polymer.

Polymer	*T_g_* °C)	Test Temperature (°C)	Test Temperature > *T*_g_?	Surface Reduction (%)	Solution Reduction (%)
S50-DCPD	88	35 ±0.3	N	-24 ± 5	9 ± 3
S70-DCPD	45	35 ± 0.3	N	74 ± 15	4 ± 0.1
S50-DVB	72	35 ±0.3	N	20 ± 5	15 ± 3
S70-DVB	52	35 ±0.3	N	53 ± 15	23 ±0.1
S30-RO	-31	3± 1	Y	75 ± 12	29 ± 2
S50-RO	-30	3 ± 1	Y	90 ± 35	40 + 5
S30-LO	-12	3± 1	Y	55 ± 1	31 ± 7
S50-LO	-12	3± 1	Y	51 ± 1	33 ± 9

## Abbreviations

DCPD: dicylcopentadiene
DIB: 1,3-diisopropenylbenzene
DSC: differential scanning calorimetry
DVB: divinylbenzene
EDS: energy-dispersive X-ray spectroscopy
FT-IR: fourier transform infrared
HAI: hospital-acquired infection
ICP-OES: inductively coupled plasma optical emission spectroscopy
NMR: nuclear magnetic resonance
SEM: scanning electron microscopy
TLC: thin layer chromatography
WCA: water contact angle
