# Supplementary material for: Antibacterial activity of inverse vulcanized polymers
Source: Biomacromolecules. Author manuscript; Available in PMC 2023 Jul 28. (PMC7614836; doi:10.1021/acs.biomac.1c01138)
Supplement: Supporting Information [file EMS181593-supplement-Supporting_Information.pdf]

SUPPORTING INFORMATION

Antibacterial activity of inverse vulcanized polymers

Romy A. Dop[1], Daniel R. Neill[2] and Tom Hasell[1*]*

[1]Department of Chemistry, University of Liverpool, Liverpool L69 7ZD, United Kingdom

[2]Department of Clinical Infection, Microbiology and Immunology, Institute of Infection, Veterinary and Ecological Sciences, University of Liverpool, Liverpool, United Kingdom

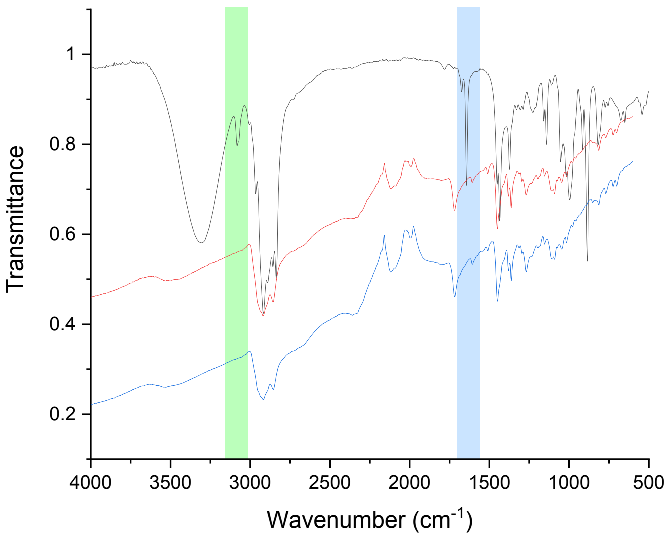

Figure S1: Fourier-transform infrared spectra (FT-IR) for perillyl alcohol (black), S50-PA (red) and S70-PA (blue). Highlighted in green at ca. 3100 cm^{-1} is the signal corresponding to =C-H stretch, and highlighted in blue at ca. 1640 cm^{-1} is the signal corresponding to an alkene C=C stretch. Both signals are visible in the spectrum of the monomer but are not visible in the spectra of the polymers suggesting alkene consumption during the reaction.

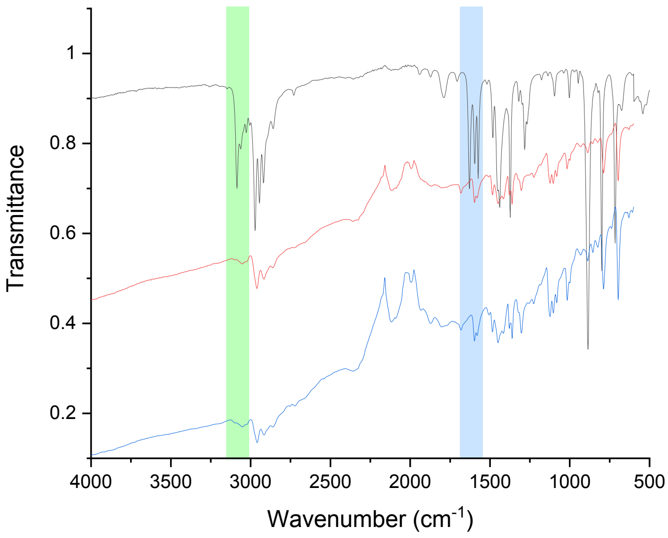

Figure S2: Fourier-transform infrared spectra (FT-IR) for DIB (black), S50-DIB (red) and S70-DIB (blue). Highlighted in green at ca. 3100 cm^{-1} is the signal corresponding to =C-H stretch, and highlighted in blue at ca. 1640 cm^{-1} is the signal corresponding to an alkene C=C stretch. Both signal intensities are reduced in the polymer spectra compared to the monomer suggesting alkene consumption during the reaction.

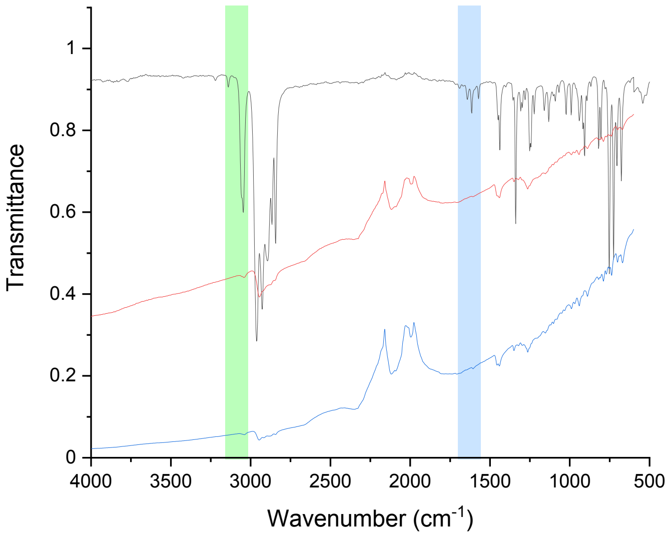

Figure S3: Fourier-transform infrared spectra (FT-IR) for DCPD (black), S50-DCPD (red) and S70-DCPD (blue). Highlighted in green at ca. 3100 cm^{-1} is the signal corresponding to =C-H stretch, and highlighted in blue at ca. 1640 cm^{-1} is the signal corresponding to an alkene C=C stretch. Both signal intensities are reduced in the polymer spectra compared to the monomer suggesting alkene consumption during the reaction.

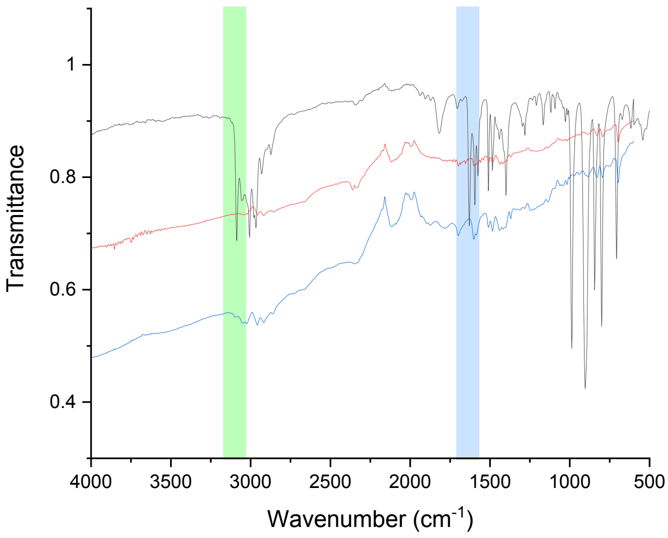

Figure S4: Fourier-transform infrared spectra (FT-IR) for DVB (black), S50-DVB (red) and S70-DVB (blue). Highlighted in green at ca. 3100 cm^{-1} is the signal corresponding to =C-H stretch, and highlighted in blue at ca. 1640 cm^{-1} is the signal corresponding to an alkene C=C stretch. Both signal intensities are reduced in the polymer spectra compared to the monomer suggesting alkene consumption during the reaction.

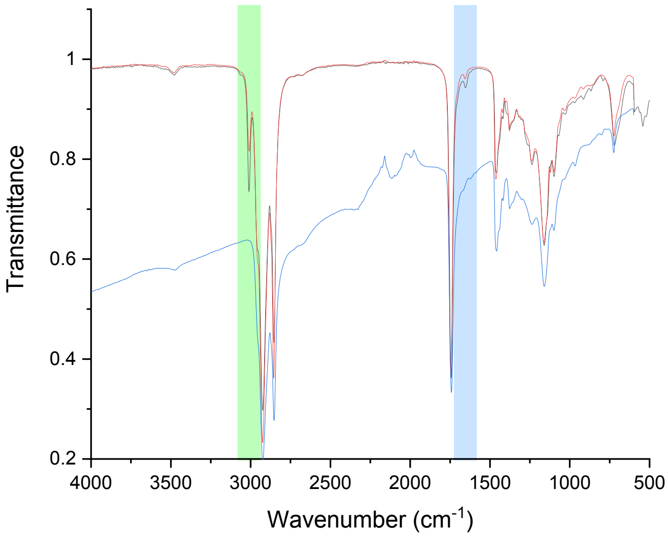

Figure S5: Fourier-transform infrared spectra (FT-IR) for rapeseed oil (black), S30-RO (red) and S50-RO (blue). Highlighted in green at ca. 3100 cm^{-1} is the signal corresponding to =C-H stretch, and highlighted in blue at ca. 1640 cm^{-1} is the signal corresponding to an alkene C=C stretch. Both signal intensities are reduced in the polymer spectra compared to the monomer suggesting alkene consumption during the reaction.

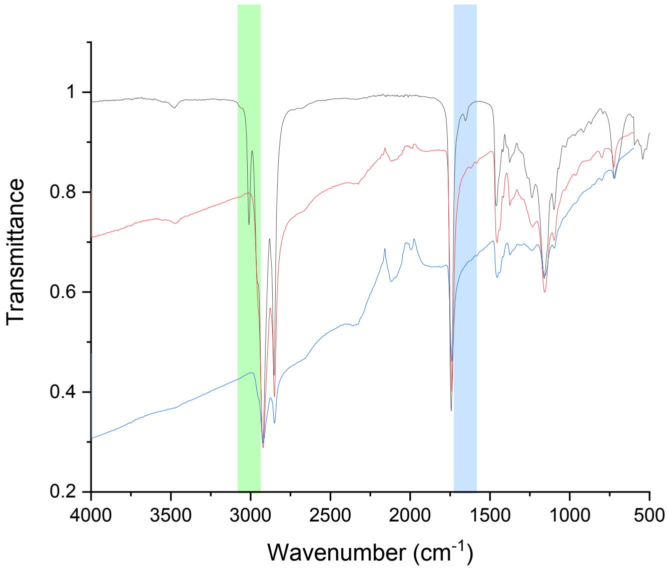

Figure S6: Fourier-transform infrared spectra (FT-IR) for linseed oil (black), S30-LO (red) and S50-LO (blue). Highlighted in green at ca. 3100 cm^{-1} is the signal corresponding to =C-H stretch, and highlighted in blue at ca. 1640 cm^{-1} is the signal corresponding to an alkene C=C stretch. Both signal intensities are reduced in the polymer spectra compared to the monomer suggesting alkene consumption during the reaction.

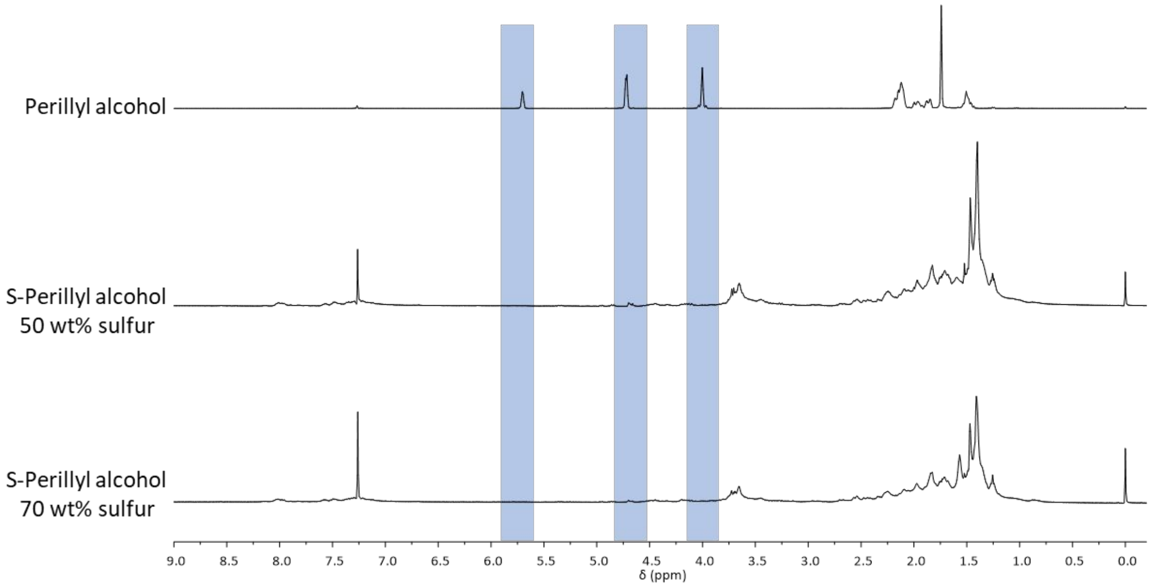

Figure S7: [1]H Nuclear magnetic resonance (NMR) spectra for perillyl alcohol oil, S50-PA and S70-PA. Highlighted are chemical shifts consistent with those of vinylic and allylic protons which are present in the spectrum for perillyl alcohol but not in the spectra of the respective polymers suggesting consumption of alkene units during reaction.

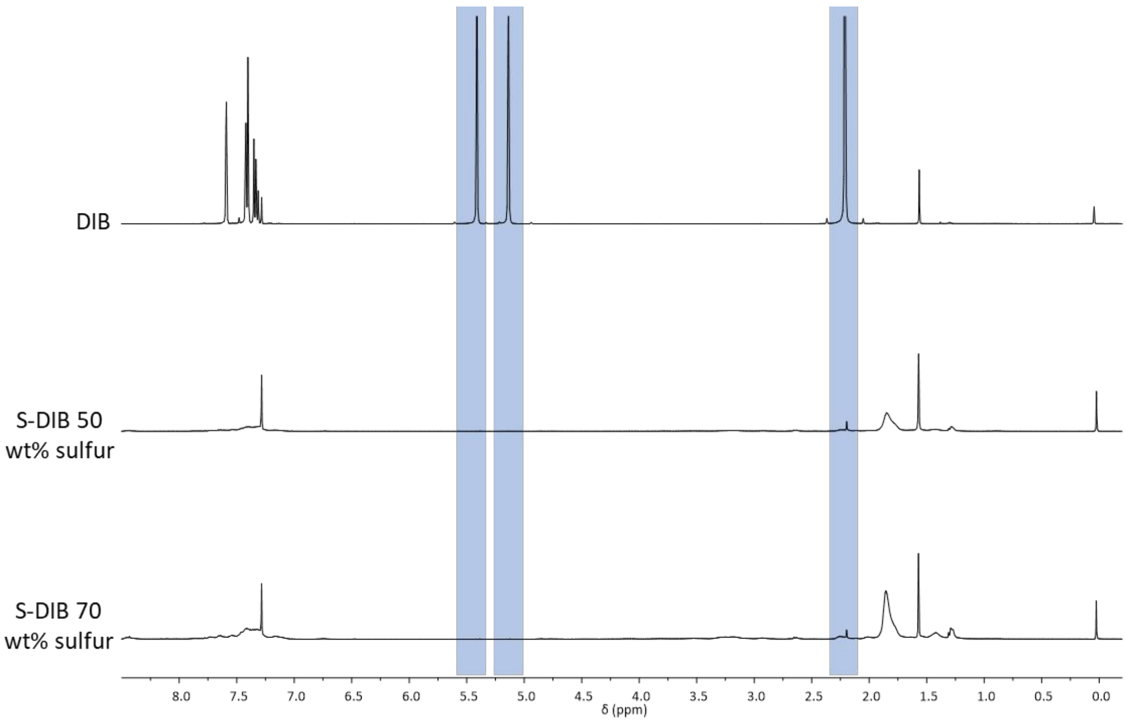

Figure S8: [1]H Nuclear magnetic resonance (NMR) spectra for DIB, S50-DIB and S70-DIB. Highlighted are chemical shifts consistent with those of vinylic (ca 5-5.5 ppm) and allylic protons (ca. 2.25 ppm) which are present in the spectrum for DIB but not in the spectra of the respective polymers suggesting consumption of alkene units during reaction.

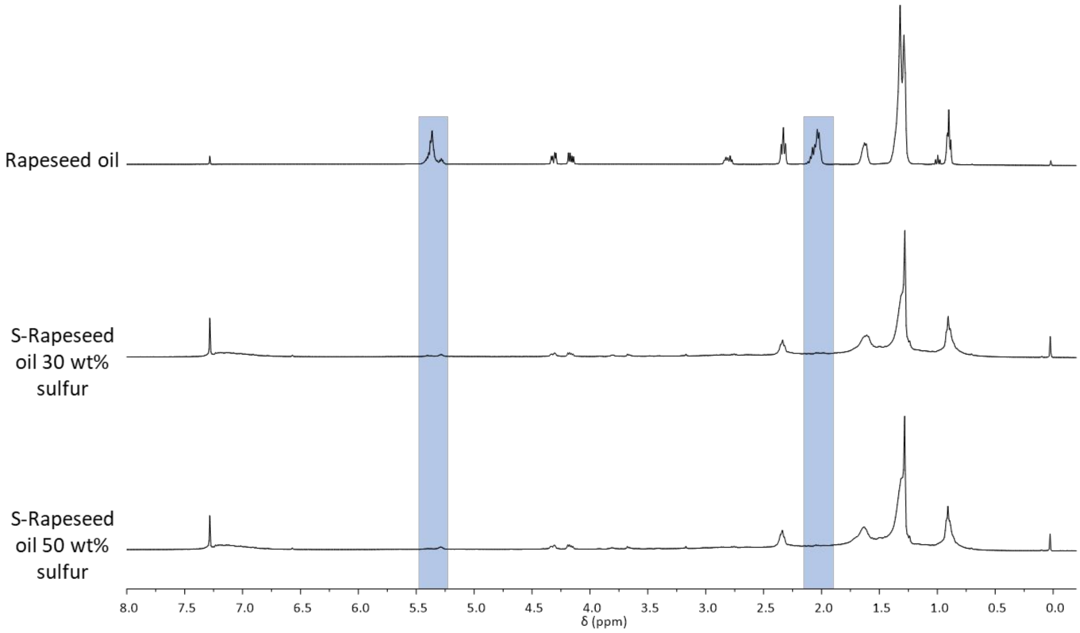

Figure S9: [1]H Nuclear magnetic resonance (NMR) spectra for rapeseed oil, S30-RO and S50-RO. Highlighted are chemical shifts consistent with those of vinylic protons (at ca. 5-5.5 ppm) and allylic protons (at ca. 1.9 ppm) which are present in the spectrum for rapeseed oil but not in the spectra of the respective polymers suggesting consumption of alkene units during reaction.

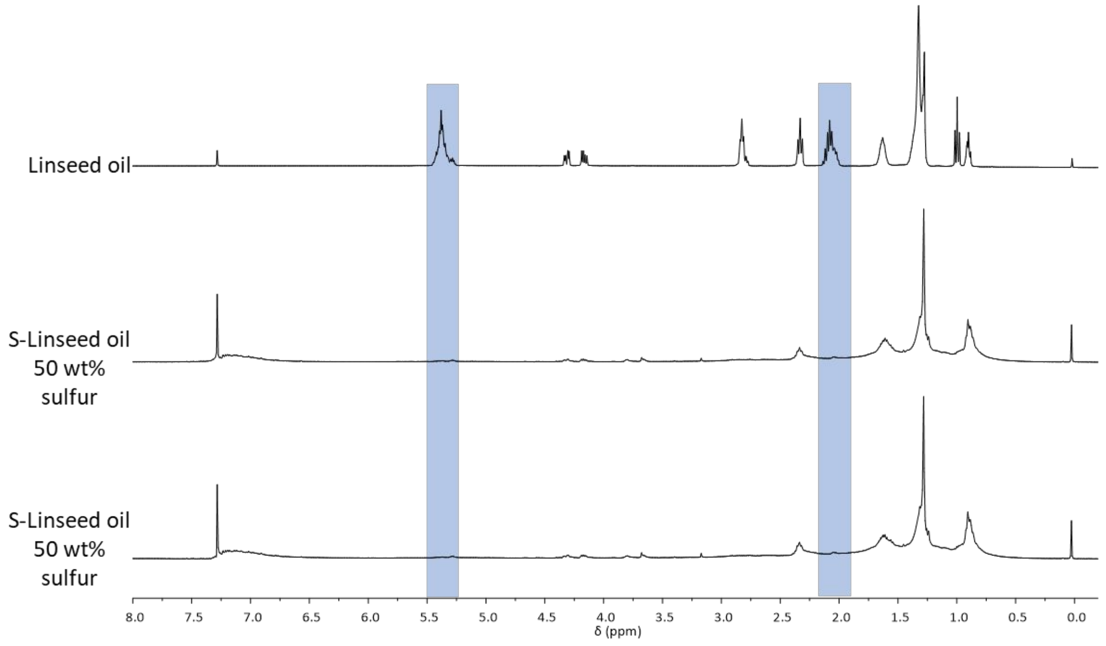

Figure S10: [1]H Nuclear magnetic resonance (NMR) spectra for linseed oil, S30-LO 30 and S50-LO. Highlighted are chemical shifts consistent with those of vinylic protons (at ca. 5-5.5 ppm) and allylic protons (at ca. 1.9 ppm) which are present in the spectrum for linseed oil but not in the spectra of the respective polymers suggesting consumption of alkene units during reaction.

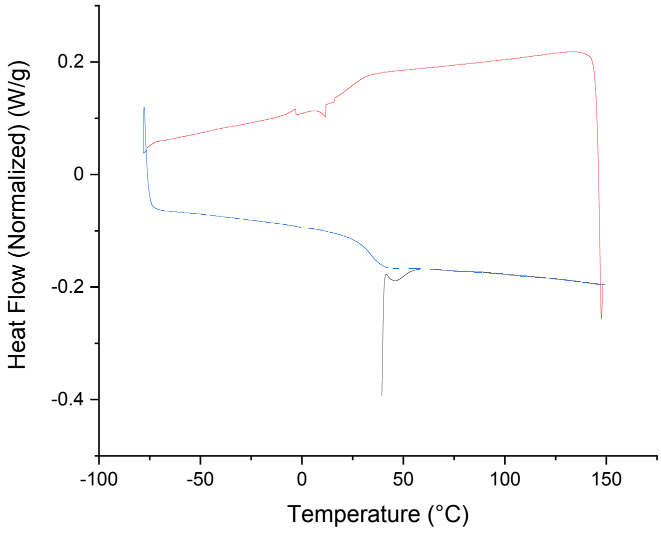

Figure S11: Differential scanning calorimetry (DSC) traces for S50-PA showing the first heating cycle to 150 °C (black), cooling to -80 °C (red), and the second heating cycle to 150 °C (blue).

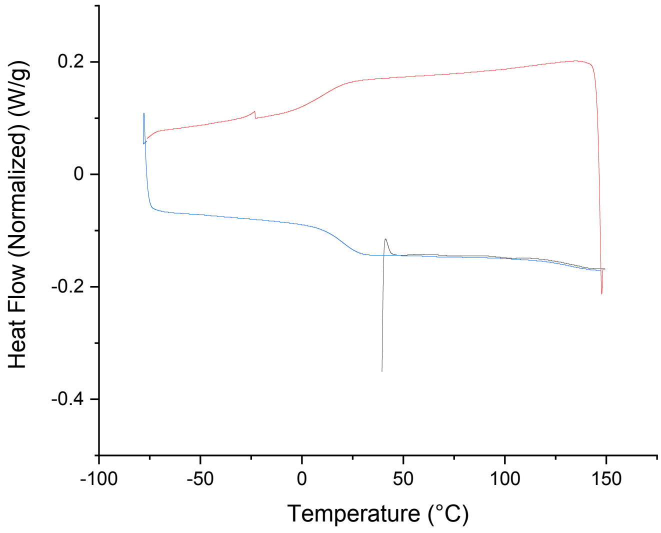

Figure S12: Differential scanning calorimetry (DSC) traces for S70-PA showing the first heating cycle to 150 °C (black), cooling to -80 °C (red), and the second heating cycle to 150 °C (blue).

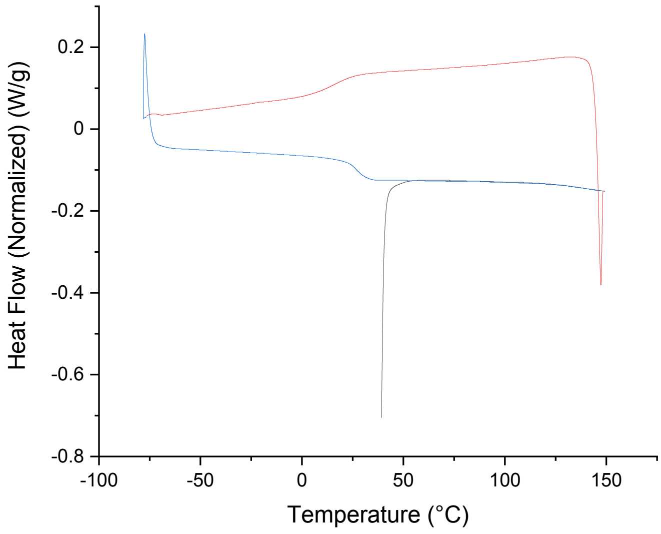

Figure S13: Differential scanning calorimetry (DSC) traces for S50-DIB showing the first heating cycle to 150 °C (black), cooling to -80 °C (red), and the second heating cycle to 150 °C (blue).

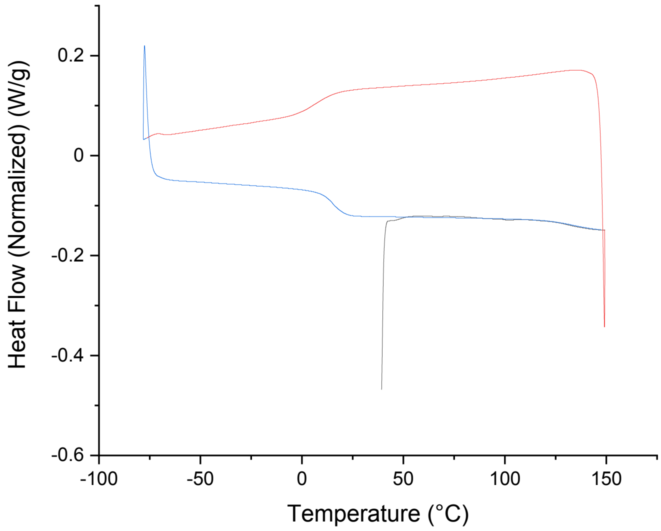

Figure S14: Differential scanning calorimetry (DSC) traces for S70-DIB showing the first heating cycle to 150 °C (black), cooling to -80 °C (red), and the second heating cycle to 150 °C (blue).

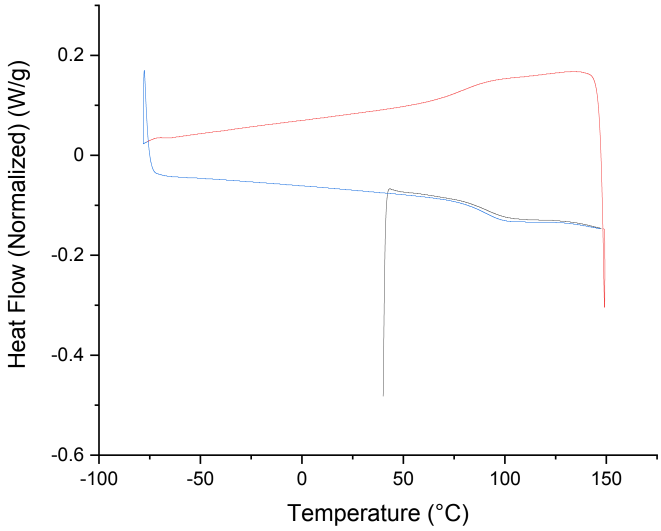

Figure S15: Differential scanning calorimetry (DSC) traces for S50-DCPD showing the first heating cycle to 150 °C (black), cooling to -80 °C (red), and the second heating cycle to 150 °C (blue).

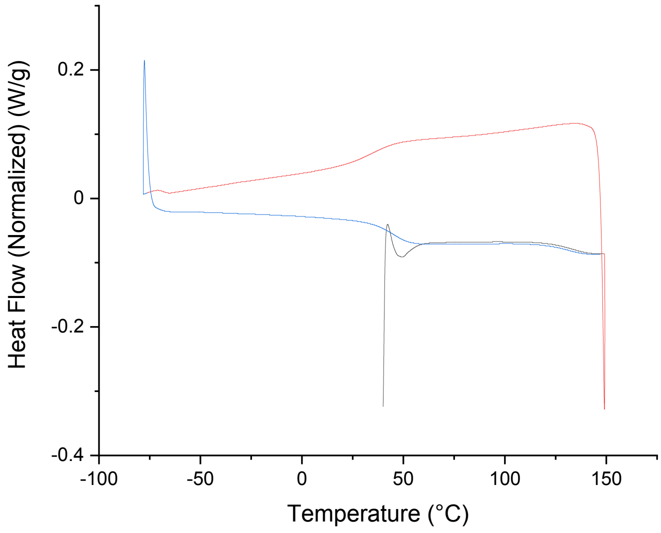

Figure S16: Differential scanning calorimetry (DSC) traces for S70-DCPD showing the first heating cycle to 150 °C (black), cooling to -80 °C (red), and the second heating cycle to 150 °C (blue).

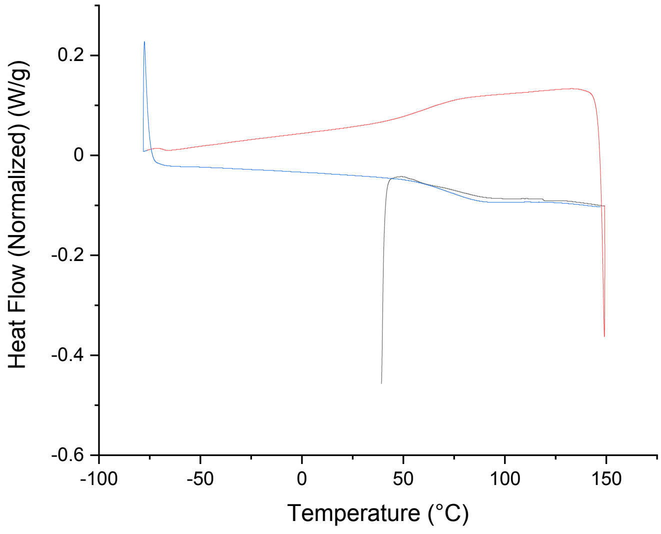

Figure S17: Differential scanning calorimetry (DSC) traces for S50-DVB showing the first heating cycle to 150 °C (black), cooling to -80 °C (red), and the second heating cycle to 150 °C (blue).

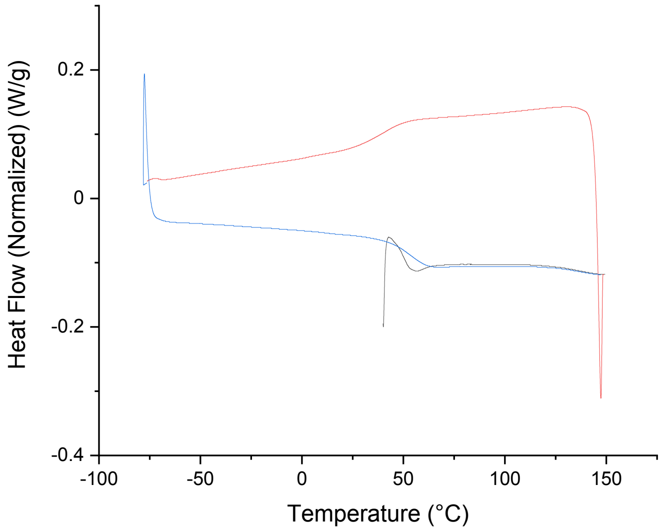

Figure S18: Differential scanning calorimetry (DSC) traces for S70-DVB showing the first heating cycle to 150 °C (black), cooling to -80 °C (red), and the second heating cycle to 150 °C (blue).

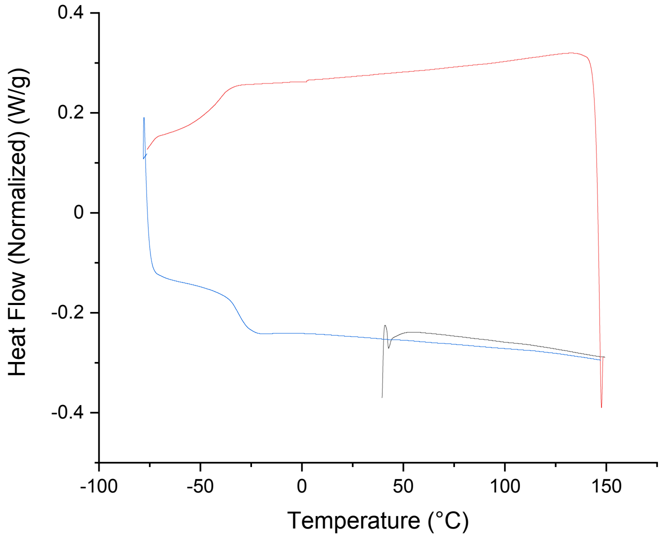

Figure S19: Differential scanning calorimetry (DSC) traces for S30-RO showing the first heating cycle to 150 °C (black), cooling to -80 °C (red), and the second heating cycle to 150 °C (blue).

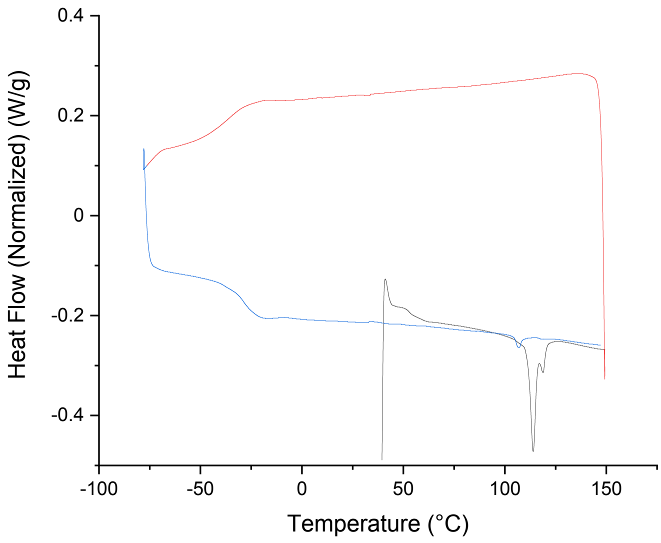

Figure S20: Differential scanning calorimetry (DSC) traces for S50-RO showing the first heating cycle to 150 °C (black), cooling to -80 °C (red), and the second heating cycle to 150 °C (blue).

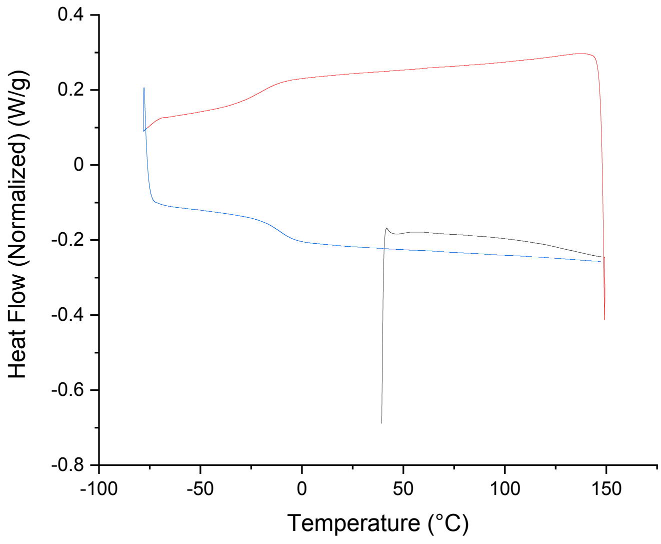

Figure S21: Differential scanning calorimetry (DSC) traces for S30-LO showing the first heating cycle to 150 °C (black), cooling to -80 °C (red), and the second heating cycle to 150 °C (blue).

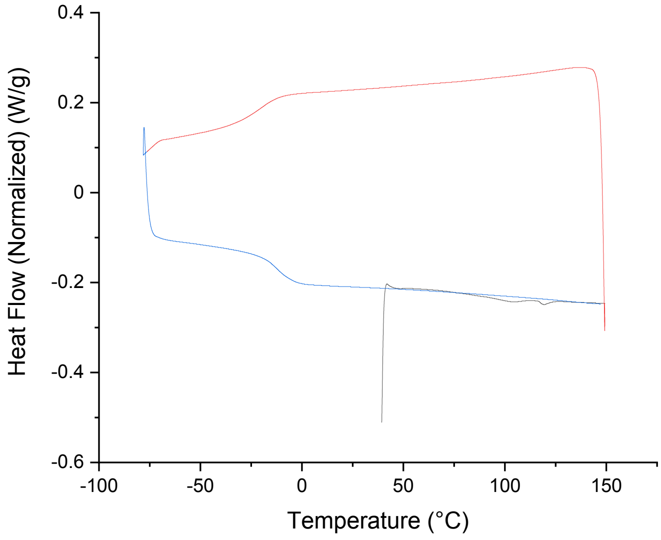

Figure S22: Differential scanning calorimetry (DSC) traces for S50-LO showing the first heating cycle to 150 °C (black), cooling to -80 °C (red), and the second heating cycle to 150 °C (blue).

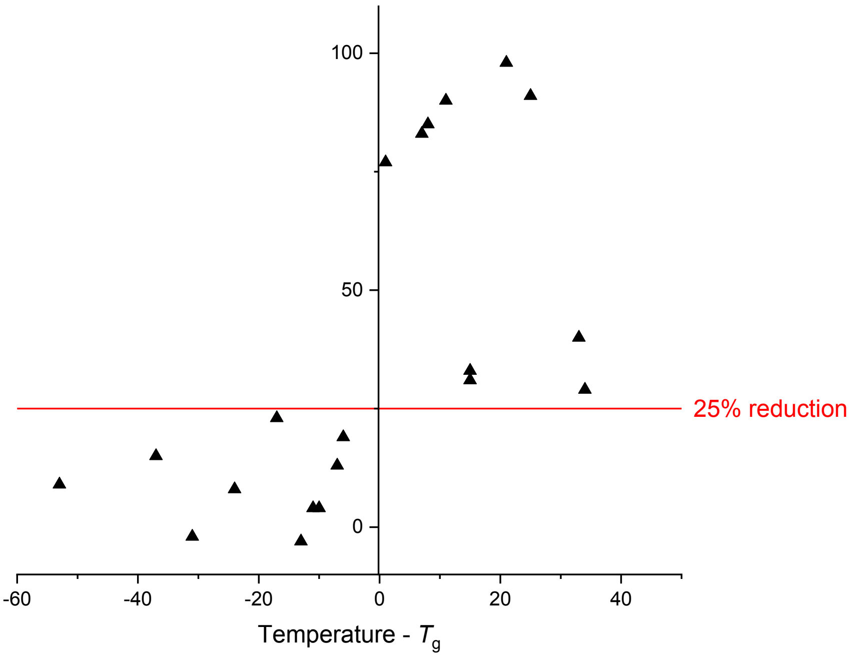

Figure S23: A plot of the % reduction in *S. aureus* cells in solution against (Temperature – T_g), where negative x axis values correspond to polymers in their glassy state and positive values correspond to polymers in the rubbery state. A greater than 25% reduction in viable cells in solution is observed for all polymers in their rubbery state.

Table S1: Table summarizing the ratios of S:PA:DCPD used to synthesize terpolymers and their calculated average sulfur rank.

Polymer	wt.% Sulfur	wt.% Perillyl alcohol	wt.% DCPD	Average sulfur rank
S50-PA	50	50	0	2.375
50:45:05	50	45	5	2.339
50:40:10	50	40	10	2.305
50:35:15	50	35	15	2.272
50:30:20	50	30	20	2.239
50:25:25	50	25	25	2.208
50:20:30	50	20	30	2.177
S50-DCPD	50	0	50	2.031

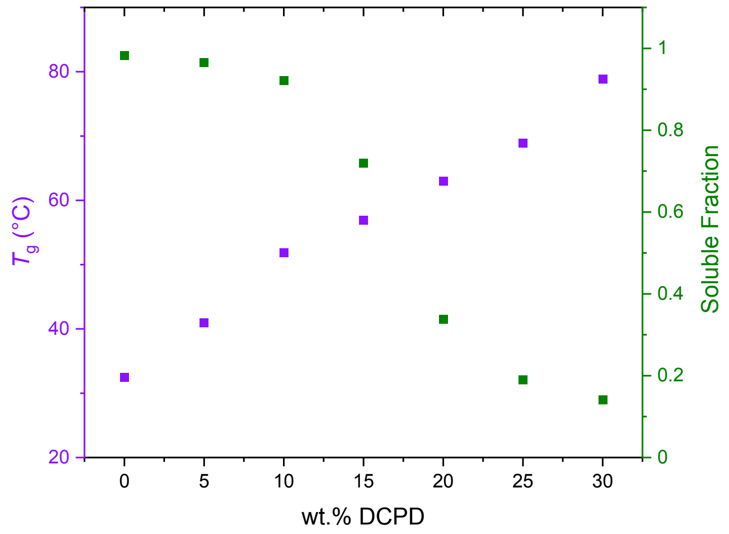

Fig S24: A plot of the T_g (°C) and soluble fraction of S-PA-DCPD terpolymers with increasing wt.% DCPD.

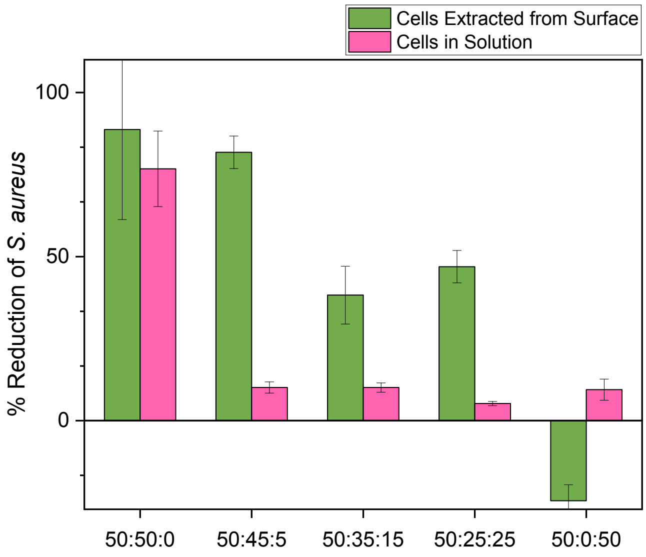

Fig S25: A summary of the % reduction in viable *S. aureus* cells extracted from the surface and from solution compared to polypropylene for S-PA-DCPD terpolymers.

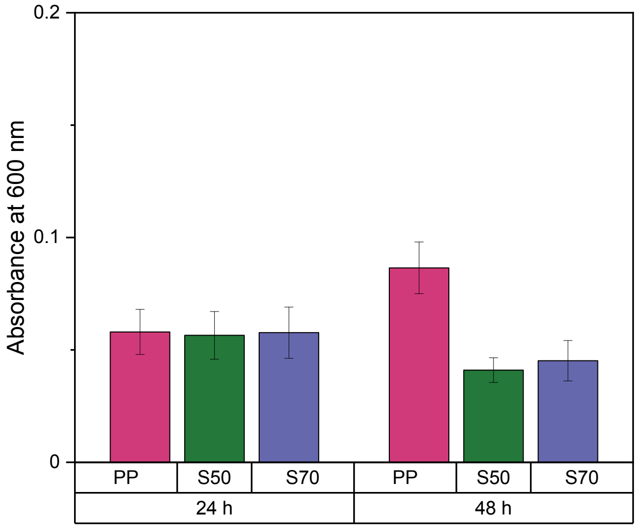

Figure S26: Absorbance at 600 nm for S-PA after staining with crystal violet after 24 and 48 h incubation at 3 °C with *S. aureus*. Where PP: Polypropylene, S50: S50-PA and S70: S70-PA.

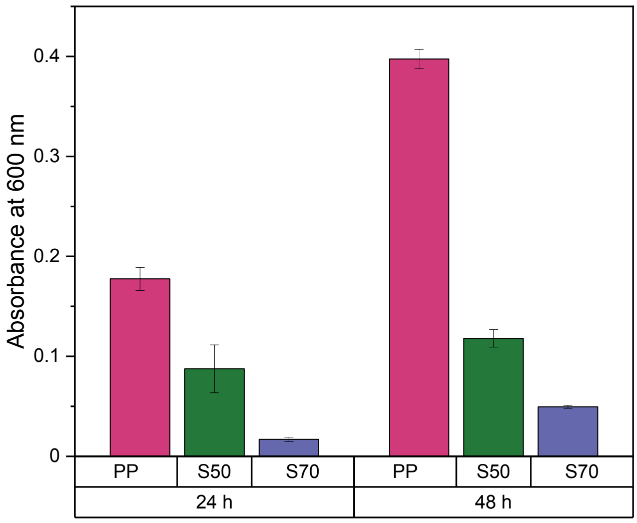

Figure S27: Absorbance at 600 nm for S-PA after staining with crystal violet after 24 and 48 h incubation at 21 °C with *S. aureus*. Where PP: Polypropylene, S50: S50-PA and S70: S70-PA.

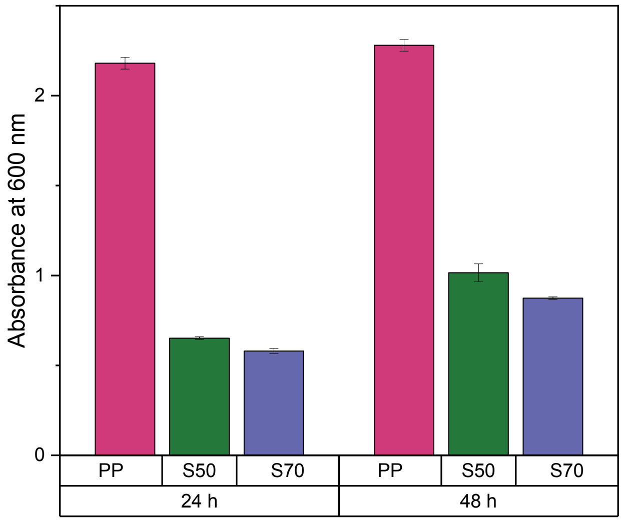

Figure S28: Absorbance at 600 nm for S-PA after staining with crystal violet after 24 and 48 h incubation at 37 °C with *P. aeruginosa*. Where PP: Polypropylene, S50: S50-PA and S70: S70-PA.

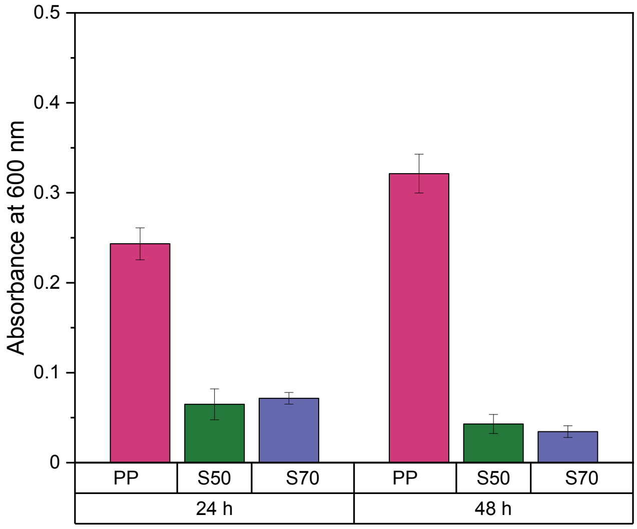

Figure S29: Absorbance at 600 nm for S-DIB after staining with crystal violet after 24 and 48 h incubation at 37 °C with *S. aureus*. Where PP: Polypropylene, S50: S50-DIB and S70: S70-DIB.

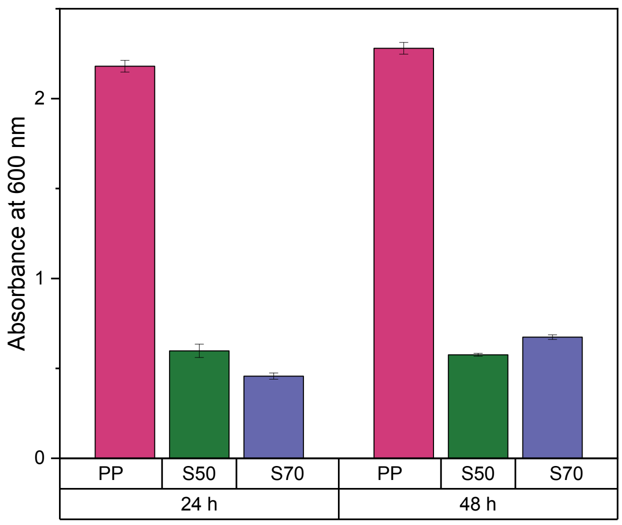

Figure S30: Absorbance at 600 nm for S-DCPD after staining with crystal violet after 24 and 48 h incubation at 37 °C with *P. aeruginosa*. Where PP: Polypropylene, S50: S50-DCPD and S70: S70-DCPD

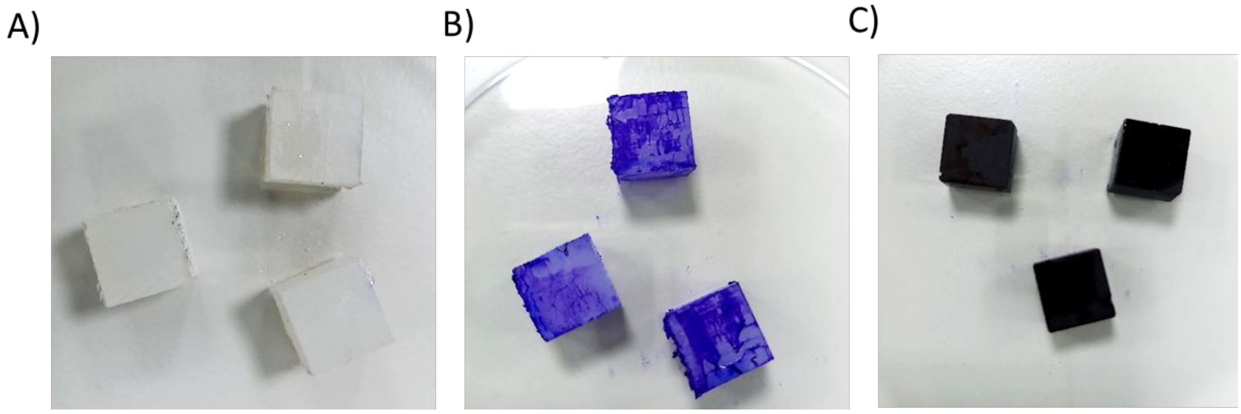

Figure S31: Image of A) clean polypropylene B) polypropylene stained with crystal violet after 48 h incubation at 37 °C with *P. aeruginosa* and C) S50-DCPD stained with crystal violet after 48 h incubation at 37 °C with *P. aeruginosa*

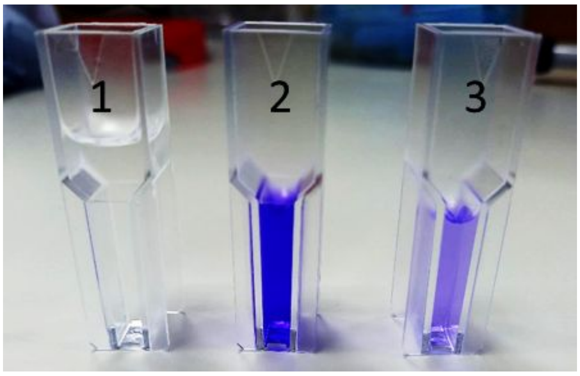

Figure S32: Image of 1) ethanol as blank solution for absorbance measurements 2) solubilized crystal violet dye from the surface of polypropylene and 3) solubilized crystal violet dye from the surface of S50-DCPD after 48 h incubation at 37 °C with *P. aeruginosa*.

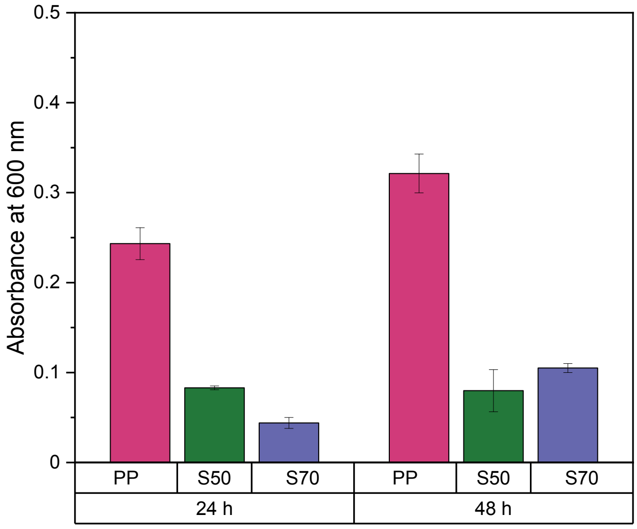

Figure S33: Absorbance at 600 nm for S-DVB after staining with crystal violet after 24 and 48 h incubation at 37 °C with *S. aureus*. Where PP: Polypropylene, S50: S50-DVB and S70: S70-DVB.

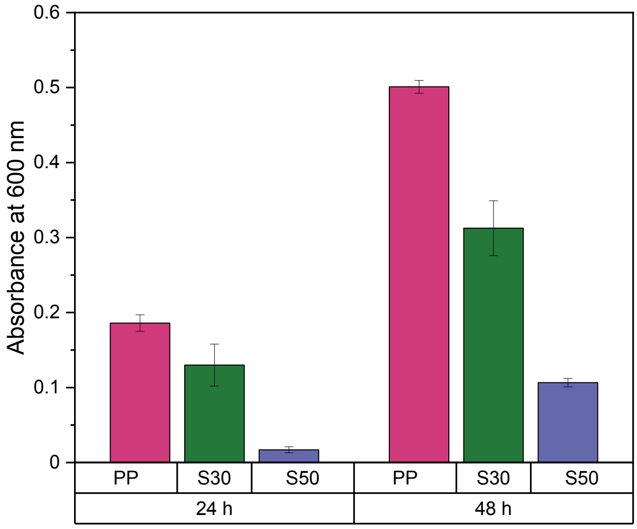

Figure S34: Absorbance at 600 nm for S-LO after staining with crystal violet after 24 and 48 h incubation at 37 °C with *S. aureus*. Where PP: Polypropylene, S30: S30-LO and S50: S50-LO.

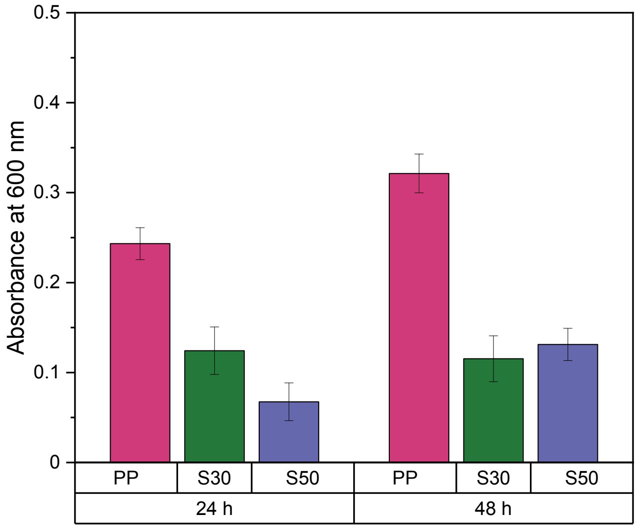

Figure S35: Absorbance at 600 nm for S-RO after staining with crystal violet after 24 and 48 h incubation at 37 °C with *S. aureus*. Where PP: Polypropylene, S30: S30-RO and S50: S50-RO.

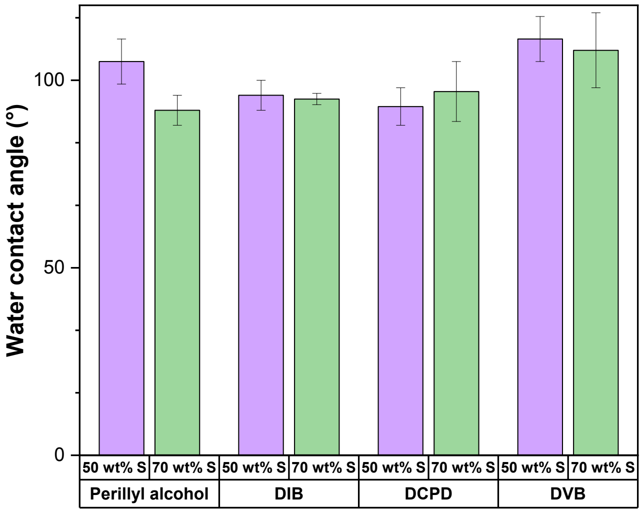

Figure S36: The water contact angle (°) of several polymers synthesised at 50 and 70 wt% sulfur measured using the sessile drop mode and Young-Laplace fitting method.

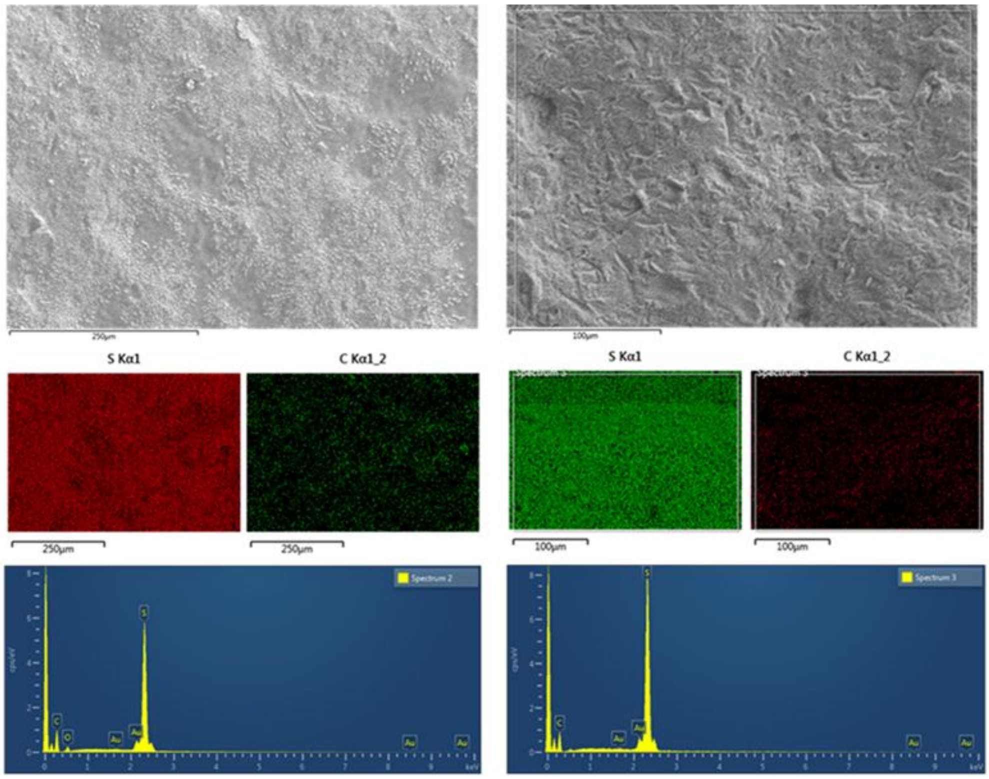

Figure S37: Scanning electron microscopy (SEM) and Energy-dispersive X-ray spectroscopy (EDS) of S50-PA (left) and S50-DCPD (right).

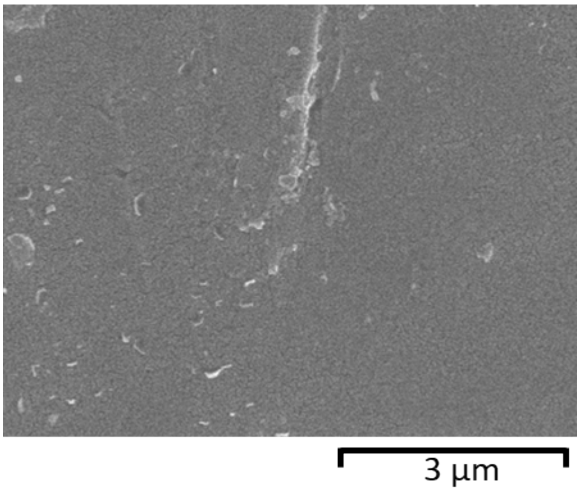

Figure S38: SEM image of clean polypropylene.

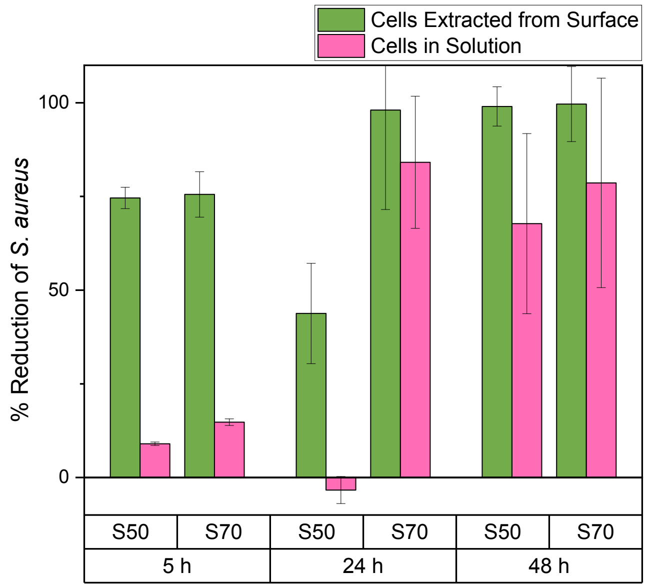

Figure S39: A summary of the % reduction in viable *S. aureus* cells extracted from the surface and from solution for S50-PA and S70-PA compared to polypropylene at 5, 24 and 48 h at room temperature.

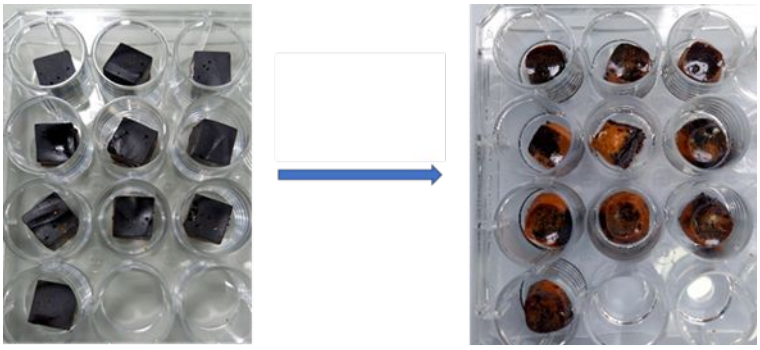

Figure S40: S70-PA before (left) and after 24 h incubation in water at 35 °C (right) for the leaching study.

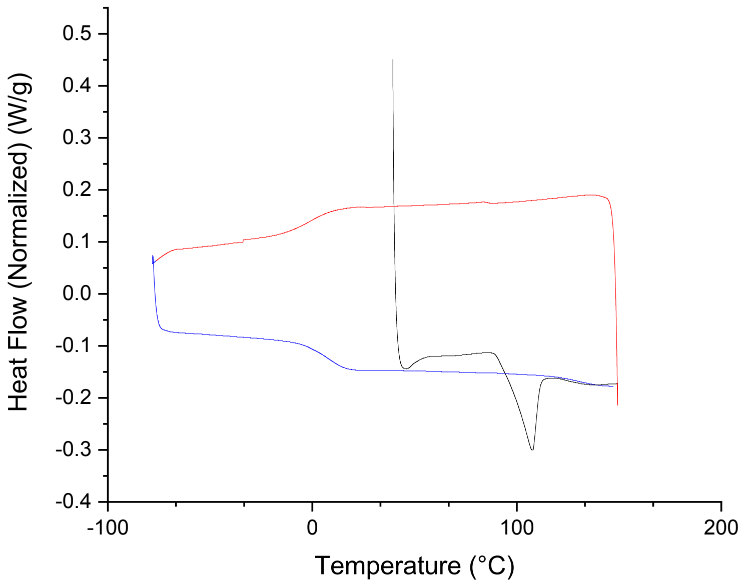

Figure S41: Differential scanning calorimetry (DSC) traces for S70-PA after 24 h incubation at 37 ºC, showing the first heating cycle to 150 °C (black), cooling to -80 °C (red), and the second heating cycle to 150 °C (blue).

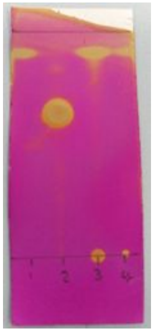

Figure S42: Thin-layer chromatography plate for S-Perillyl alcohol analysis in order to detect any unreacted perillyl alcohol and/or elemental sulfur where 1: elemental sulfur, 2: perillyl alcohol, 3: S50-PA 4: S70-PA using 1:1 hexane:ethyl acetate as the eluent.
